# Preclinical evaluation of 5-methyltetrahydrofolate-based radioconjugates—new perspectives for folate receptor–targeted radionuclide therapy

**DOI:** 10.1007/s00259-020-04980-y

**Published:** 2020-10-15

**Authors:** Patrycja Guzik, Martina Benešová, Magdalena Ratz, Josep M. Monné Rodríguez, Luisa M. Deberle, Roger Schibli, Cristina Müller

**Affiliations:** 1 Center for Radiopharmaceutical Sciences ETH-PSI-USZ, Paul Scherrer Institute - PSI, 5232 Villigen-PSI, Switzerland; 2 Department of Chemistry and Applied Biosciences, ETH Zurich, 8093 Zurich, Switzerland; 3 Laboratory for Animal Model Pathology (LAMP), Institute of Veterinary Pathology, Vetsuisse Faculty, University of Zurich, 8057 Zurich, Switzerland

**Keywords:** Folate receptor, 5-Methyltetrahydrofolate, Radionuclide therapy, Lutetium-177, Albumin-binding entity

## Abstract

**Purpose:**

The folate receptor (FR) is frequently overexpressed in a variety of tumor types and, hence, an interesting target for radionuclide therapy. The aim of this study was to evaluate a new class of albumin-binding radioconjugates comprising 5-methyltetrahydrofolate (5-MTHF) as a targeting agent and to compare their properties with those of the previously established folic acid-based [^177^Lu]Lu-OxFol-1.

**Methods:**

[^177^Lu]Lu-6*R*-RedFol-1 and [^177^Lu]Lu-6*S*-RedFol-1 were investigated in vitro using FR-positive KB tumor cells. Biodistribution studies were performed in KB tumor-bearing mice, and the areas under the curve (AUC_0 → 120h_) were determined for the uptake in tumors and kidneys. [^177^Lu]Lu-6*R*-RedFol-1 was compared with [^177^Lu]Lu-OxFol-1 in a therapy study over 8 weeks using KB tumor-bearing mice.

**Results:**

Both radioconjugates demonstrated similar in vitro properties as [^177^Lu]Lu-OxFol-1; however, the tumor uptake of [^177^Lu]Lu-6*R*-RedFol-1 and [^177^Lu]Lu-6*S*-RedFol-1 was significantly increased in comparison with [^177^Lu]Lu-OxFol-1. In the case of [^177^Lu]Lu-6*S*-RedFol-1, also the kidney uptake was increased; however, renal retention of [^177^Lu]Lu-6*R*-RedFol-1 was similar to that of [^177^Lu]Lu-OxFol-1. This led to an almost 4-fold increased tumor-to-kidney AUC_0 → 120h_ ratio of [^177^Lu]Lu-6*R*-RedFol-1 as compared with [^177^Lu]Lu-6*S*-RedFol-1 and [^177^Lu]Lu-OxFol-1. At equal activity, the therapeutic effect of [^177^Lu]Lu-6*R*-RedFol-1 was better than that of [^177^Lu]Lu-OxFol-1, reflected by a slower tumor growth and, consequently, an increased median survival time (49 days vs. 34 days).

**Conclusion:**

This study demonstrated the promising potential of 5-MTHF-based radioconjugates for FR-targeting. Application of [^177^Lu]Lu-6*R*-RedFol-1 resulted in unprecedentedly high tumor-to-kidney ratios and, as a consequence, a superior therapeutic effect as compared with [^177^Lu]Lu-OxFol-1. These findings, together with the absence of early side effects, make [^177^Lu]Lu-6*R*-RedFol-1 attractive in view of a future clinical translation.

**Electronic supplementary material:**

The online version of this article (10.1007/s00259-020-04980-y) contains supplementary material, which is available to authorized users.

## Introduction

Targeted radionuclide therapy emerged as a promising concept for the palliative treatment of metastasized cancer using β^−^-particle-emitting radionuclides in combination with a specific tumor-targeting agent [1, 2]. The experience made with somatostatin receptor-targeted radiopeptides (e.g., [^177^Lu]Lu-DOTATATE [3, 4]) and prostate-specific membrane antigen (PSMA)-targeted radioligands (e.g., [^177^Lu]Lu-PSMA-617 [5, 6]) is encouraging to further explore suitable targets and develop radiopharmaceuticals to enable the treatment of additional tumor types.

The folate receptor (FR) is a membrane-anchored glycoprotein, which is overexpressed in gynecological and other tumor types, including lung, breast, and colon cancers [7–9]. Folic acid radioconjugates have been translated to clinics for nuclear imaging of FR-positive tumors [10, 11]; however, their therapeutic exploitation remains challenging. The relatively low tumor-to-kidney ratio of accumulated folate radioconjugates would limit the applicable quantity of activity in order to prevent the risk of damage to the kidneys [12].

The high renal uptake of folate-based radiopharmaceuticals has been addressed with various strategies [13], including pharmacological interactions [14–16]. A major step forward was achieved by introducing an albumin-binding entity into the structure of radiofolates to prolong their blood circulation [17, 18]. The resulting radioconjugates revealed significantly increased tumor uptake and improved tumor-to-kidney ratios, which enabled their use in preclinical therapy studies in mice [17, 19]. The therapeutic effects of this approach were promising; however, the kidneys remained the dose-limiting organ.

Recently, Boss et al. reported on results obtained with a novel class of fluorine-18-based radiotracers, in which folic acid (oxidized version of folate) was exchanged with the two stereoisomers (6*R* and 6*S*, respectively) of 5-methyltetrahydrofolate (5-MTHF) [20, 21]. It was found that 6*R*-aza-[^18^F]fluoro-5-MTHF as well as 6*S*-aza-[^18^F]fluoro-5-MTHF accumulated to a significantly higher extent in the tumor tissue than aza-[^18^F]fluorofolic acid (^18^F-AzaFol), in which folic acid was employed as a targeting agent [21]. Importantly, the 6*R*-aza-[^18^F]fluoro-5-MTHF isomer was cleared much more effectively through the kidneys as compared with ^18^F-AzaFol.

**Fig. 1:**
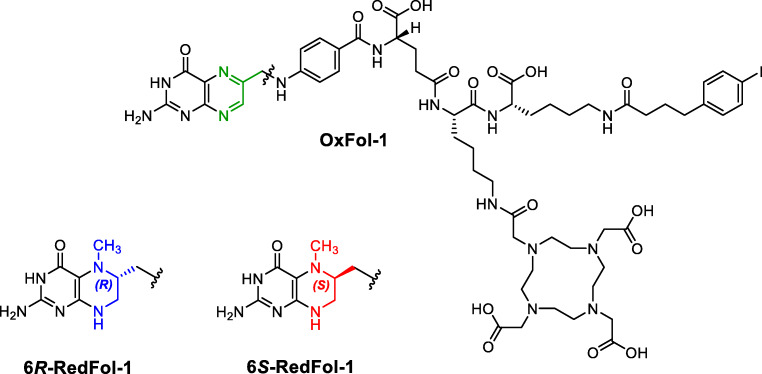
Chemical structure of OxFol-1 (green), 6*R*-RedFol-1 (blue), and 6*S*-RedFol-1 (red)

Thus, the aim of this study was to translate the concept of using 5-MTHF as a targeting agent to albumin-binding DOTA conjugates in order to increase the tumor uptake and possibly reduce renal retention of activity. 6*R*-RedFol-1 and 6*S*-RedFol-1, based on 6*R*-5-MTHF and 6*S*-5-MTHF, respectively, were designed as structural equivalents to the previously developed albumin-binding DOTA-folate conjugate (cm10, herein referred to as OxFol-1 [18]) (Fig. 1). 6*R*-RedFol-1 and 6*S*-RedFol-1 were labeled with lutetium-177 and evaluated in vitro and in vivo for comparison of their characteristics with those of [^177^Lu]Lu-OxFol-1 [18]. Therapy studies with KB tumor-bearing mice were performed in order to compare the therapeutic effect of the more promising [^177^Lu]Lu-RedFol-1 isomer with [^177^Lu]Lu-OxFol-1.

## Materials and methods

### Radiolabeling and in vitro stability of the folate radioconjugates

The synthesis of 6*R*-RedFol-1 and 6*S*-RedFol-1 will be published elsewhere. OxFol-1 (previously referred to as cm10) was employed in previous preclinical studies [13, 22]. The radiolabeling of the folate conjugates with lutetium-177 (*T*_1/2_ = 6.65 days, ${E}_{{\boldsymbol{\upbeta}}^{-}\mathrm{average}}$= 134 keV, *E*_γ_ = 113 keV, 208 keV) was performed at pH ~ 4.5 using no-carrier-added lutetium-177 ([^177^Lu]LuCl_3_ in HCl 0.04 M; ITM Medical Isotopes GmbH, Germany) to obtain molar activities of 10–50 MBq/nmol (Supplementary Material). l-Ascorbic acid (6 mg) was added to the labeling mixture of 6*R*-RedFol-1 and 6*S*-RedFol-1 to prevent oxidation. Quality control was performed using high-performance liquid chromatography (HPLC). Stability of the radioconjugates (50 MBq/nmol) in phosphate-buffered saline (PBS) pH 7.4 at an activity concentration of 100 MBq/500 μL was investigated over a period of 24 h (Supplementary Material). Stability in human plasma (Stiftung Blutspende SRK Aargau-Solothurn, Switzerland) was also investigated over 24 h at 37 °C (Supplementary Material).

### Determination of logD values

Distribution coefficients (logD values) of the folate radioconjugates were determined by a shake-flask method using *n*-octanol and PBS pH 7.4 followed by phase separation, as previously reported (Supplementary Material) [18].

### Binding affinity to mouse and human plasma proteins

To compare the plasma protein-binding properties of the folate radioconjugates, the percentage of the fraction bound to mouse and human plasma proteins was determined at variable plasma dilutions calculated as [albumin]-to-[radioconjugate] molar ratios. Determination of the binding affinity was performed by measuring the free fraction of the radioconjugate separated from the albumin-bound fraction using an ultrafiltration method as previously reported (Supplementary Material) [23].

### Tumor cell culture and cell uptake studies

KB tumor cells (cervical carcinoma cell line, subclone of HeLa cells, ACC-136) were purchased from the German Collection of Microorganisms and Cell Cultures (DSMZ, Germany). Cells were cultured in folate-deficient RPMI medium (FFRPMI, Cell Culture Technologies GmbH, Gravesano, Switzerland) supplemented with 10% fetal calf serum, l-glutamine, and antibiotics.

Cellular uptake and internalization studies were performed with all folate radioconjugates according to a previously published procedure (Supplementary Material) [18]. The results were expressed as percentage of total added activity and presented as average ± SD of 3–6 independent experiments performed in triplicate.

### In vivo studies

All applicable international, national, and/or institutional guidelines for the care and use of animals were followed. In particular, all animal experiments were carried out according to the guidelines of Swiss Regulations for Animal Welfare. The preclinical studies have been ethically approved by the Cantonal Committee of Animal Experimentation and permitted by the responsible cantonal authorities.

Five- to 6-week-old female, athymic nude mice (CD-1 Foxn-1/nu) were purchased from Charles River Laboratories (Sulzfeld, Germany) and fed with a folate-deficient rodent diet (ssniff Spezialdiäten GmbH; Soest, Germany). Mice were subcutaneously inoculated with KB tumor cells (5 × 10^6^ cells in 100 μL PBS) on both shoulders for biodistribution and imaging studies or with KB tumor cells (4.5 × 10^6^ cells in 100 μL PBS) on the right shoulder for the therapy study.

### In vivo stability of folate radioconjugates

In vivo stability studies were performed in mice without tumors (*n* = 2), injected with the folate radioconjugates (25 MBq, 0.5 nmol, 100 μL). After precipitation of proteins in plasma of blood samples collected at 4 h p.i., the samples were analyzed using HPLC (Supplementary Material).

### Biodistribution studies

Biodistribution studies were performed 10–14 days after tumor cell inoculation when the tumor size reached a volume of ~ 300 mm^3^. Mice (*n* = 4) were injected into a lateral tail vein with the respective folate radioconjugate (3 MBq, 0.5 nmol, 100 μL) diluted in PBS containing 0.05% bovine serum albumin (BSA). The animals were sacrificed at various timepoints up to 120 h after administration of the radioconjugates. Additional mice (*n* = 3) were injected with excess folic acid (100 μg in PBS pH 7.4), ~ 5 min prior to the folate radioconjugates and sacrificed 1 h later (Supplementary Material). Selected tissues and organs were collected, weighed, and counted using a γ-counter (PerkinElmer, Wallac Wizard 1480). The results were listed as a percentage of the injected activity per gram (% IA/g) of tissue mass, using counts of a defined volume of the original injection solution measured at the same time, resulting in decay-corrected values.

### Determination of areas under the curve

Biodistribution data were converted to non-decay-corrected values to obtain the time-dependent curves of accumulated activity in the tumor xenografts, blood, kidneys, and liver. The data points were used to calculate the areas under the curve (AUC) using GraphPad Prism (version 7) as previously reported [18]. The AUC_0 → 120h_ values of the [^177^Lu]Lu-OxFol-1 were set as 1.0 to determine the relative values of [^177^Lu]Lu-6*R*-RedFol-1 and [^177^Lu]Lu-6*S*-RedFol-1, respectively.

### SPECT/CT imaging studies

The acquisition and analysis of images were performed with a dedicated small-animal SPECT/CT scanner (NanoSPECT/CT™, Mediso Medical Imaging Systems, Budapest, Hungary) as previously reported (Supplementary Material) [18]. Mice were injected with the folate radioconjugates (25 MBq, 0.5 nmol) and scanned at 4 h and 24 h post injection (p.i.) Images were prepared using VivoQuant post-processing software (version 3.5, inviCRO Imaging Services and Software, Boston, USA). A Gauss post-reconstruction filter (FWHM = 1 mm) was applied, and the scale of activity was set as indicated on the images.

### Therapy study

Mice were randomly assigned to five groups consisting of 6–9 animals 4 days after tumor cell inoculation when tumors reached an average size of 60–100 mm^3^. The mice were injected with vehicle only (group A: PBS with 0.05% BSA; control), 10 MBq [^177^Lu]Lu-6*R*-RedFol-1 (group B), or 10 MBq [^177^Lu]Lu-OxFol-1 (group C) at an amount of 0.5 nmol folate conjugate. Additional groups of mice were injected with 15 MBq [^177^Lu]Lu-6*R*-RedFol-1 (group D) or 15 MBq [^177^Lu]Lu-OxFol-1 (group E) at an amount of 0.5 nmol folate conjugate (Table 1). The relative body weight (RBW) was defined as [BW_*x*_/BW_0_], where BW_*x*_ is the body weight in gram at a given day *x* and BW_0_ is the body weight in gram at day 0. The tumor dimensions were determined by measuring the longest tumor axis (*L*) and its perpendicular axis (*W*) with a digital caliper. The tumor volume (TV) was calculated according to the equation [TV = 0.5 × (*L* × *W*^2^)]. The relative tumor volume (RTV) was defined as [TV_*x*_/TV_0_], where TV_*x*_ is the tumor volume in cubic millimeters at a given day *x* and TV_0_ is the tumor volume in cubic millimeters at day 0. Endpoint criteria were defined as (i) a tumor volume of ≥ 1000 mm^3^, (ii) loss of ≥ 15% of initial body weight, (iii) a combination of a tumor size of ≥ 800 mm^3^ and body weight loss of ≥ 10% and/or (iv) ulceration of the tumor, and/or (v) abnormal behavior, indicating pain or unease. Mice were removed from the study and euthanized when an endpoint was reached.

**Table 1:** Design of the therapy study including the average tumor volumes and body weights of mice at therapy start

Group	Treatment	Number of mice	Injected activity and molar amount	Tumor volume^2^ (mm^3^) (average ± SD)	Body weight (g) (average ± SD)
				Day 0	Day 0
A	Vehicle^1^	9	-	84 ± 24	23.7 ± 2.7
B	[^177^Lu]Lu-6*R*-RedFol-1	6	10 MBq, 0.5 nmol	66 ± 8	24.6 ± 1.2
C	[^177^Lu]Lu-OxFol-1	6	10 MBq, 0.5 nmol	69 ± 22	24.9 ± 1.2
D	[^177^Lu]Lu-6*R*-RedFol-1	6	15 MBq, 0.5 nmol	99 ± 33	20.0 ± 0.8^3^
E	[^177^Lu]Lu-OxFol-1	6	15 MBq, 0.5 nmol	95 ± 20	20.9 ± 1.2^3^

^1^Vehicle: 0.05% BSA in PBS

^2^No significant differences determined between the tumor volumes measured for each group (*p* > 0.05);

^3^Significantly lower body weights compared with mice of groups A, B, and C

### Assessment of the therapy study

The efficacy of the radionuclide therapy was assessed by determination of the tumor growth delay (TGD_*x*_), which was calculated as the time required for the tumor volume to increase *x*-fold over the initial volume at day 0. The tumor growth delay indices [TGDI_*x*_ = TGD_*x*_(T)/TGD_x_(C)] were calculated as the TGD_x_ ratio of treated mice (T) over control mice (C) for a 2-fold (*x* = 2, TGD_2_) and 5-fold (*x* = 5, TGD_5_) increase of the initial tumor volume. The percentage of tumor growth inhibition (TGI) was calculated as [100 − (RTV_T_/RTV_C_ × 100)], where RTV_T_ is a relative tumor volume of treated mice at day 14, when the first mouse of the control group (group A) reached the endpoint and the average relative tumor volume of control mice was RTV_C_.

The average of relative body weights of mice from each group was compared with that of control mice at day 14 and at the endpoint. Blood plasma parameters were determined once an endpoint was reached or at the end of the study (Supplementary Material). After euthanasia, the kidneys, liver, spleen, and brain were collected and weighed. The organ mass-to-brain ratios were calculated using the organ masses collected at the day of euthanasia (Supplementary Material).

A full macroscopic examination was performed in each animal, and the kidneys, bone marrow (sternum and femur), and spleen were sampled for histological assessment as previously reported (Supplementary Material) [24]. Histological lesions were semi-quantitatively scored by a veterinary pathologist in a blind manner using a severity grading scheme that ranged from 0 to 5.

### Statistical analysis and figure preparation

**Fig. 2:**
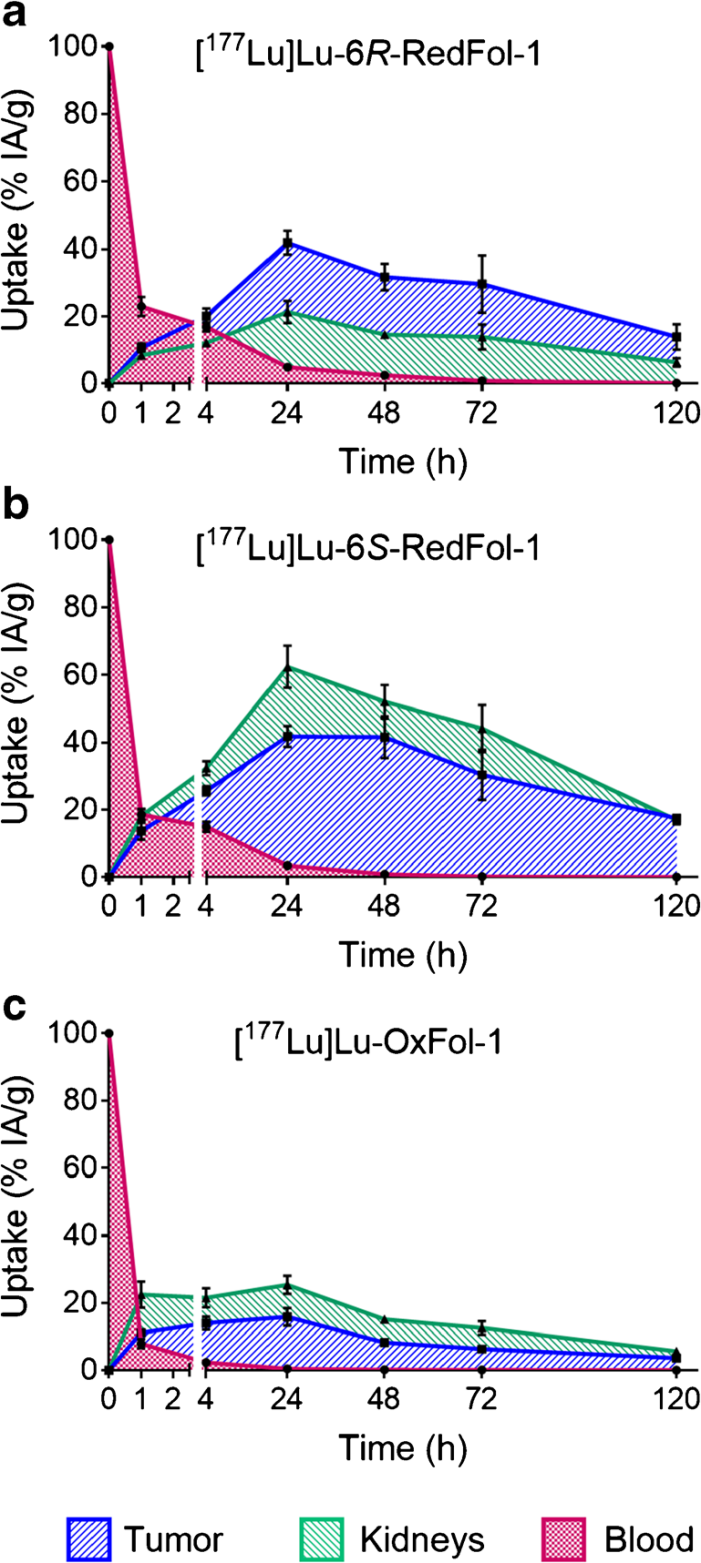
Graphs representing the AUC_0 → 120h_ of non-decay-corrected biodistribution data up to 120 h p.i. of the folate radioconjugates. **a** [^177^Lu]Lu-6*R*-RedFol-1. **b** [^177^Lu]Lu-6*S*-RedFol-1. **c** [^177^Lu]Lu-OxFol-1 (adapted with permission from Siwowska et al. 2017, Mol Pharm 14:523 [18]. Copyright 2020 American Chemical Society). Each data point represents the average of a group of mice ± SD (*n* = 4)

Binding affinity to plasma proteins was statistically analyzed using one-way ANOVA with Dunnett’s multiple comparisons post-test. Analyses of biodistribution data and the absolute AUC_0 → 120h_ values were performed with two-way or one-way ANOVA with Tukey’s multiple comparisons post-test. The therapy study was analyzed for significance using a one-way ANOVA with Tukey’s or Dunnett’s test. Survival of mice was assessed using Kaplan-Meier curves to determine median survival of mice of each group. All analyses were performed using GraphPad Prism program (version 7.0). A *p* value of < 0.05 was considered statistically significant. Graphs of Figs. 2 and 4 were prepared using GraphPad Prism software (version 7).

## Results

### Radiolabeling, stability, and logD values of ^177^Lu-folate conjugates

Radiolabeling with lutetium-177 was readily achieved to obtain the folate radioconjugates at a radiochemical purity of > 99% (Supplementary Material, Fig. S1). All folate radioconjugates were stable in PBS at room temperature (> 97% intact radioconjugates) over 24 h (Supplementary Material, Table S1). High stability was also determined after incubation of the folate radioconjugates in human plasma (≥ 98% intact radioconjugates) over 24 h at 37 °C. The logD values of [^177^Lu]Lu-6*R*-RedFol-1 (− 3.92 ± 0.11) and [^177^Lu]Lu-6*S*-RedFol-1 (− 3.70 ± 0.10) were slightly higher than the logD value of [^177^Lu]Lu-OxFol-1 (− 4.21 ± 0.14).

### Binding affinity to mouse and human plasma proteins

At a physiological albumin concentration, represented by the highest [mouse serum albumin (MSA)]-to-[radioconjugate] molar ratio used in this study, ~ 93% of [^177^Lu]Lu-6*R*-RedFol-1 and [^177^Lu]Lu-6*S*-RedFol-1 were bound to plasma proteins (Supplementary Material, Fig. S2a). Under the same experimental conditions, the plasma-bound fraction of [^177^Lu]Lu-OxFol-1 was slightly lower (~ 90%). The binding at the corresponding molar ratio of human serum albumin (HSA) was higher for all three radioconjugates, but in analogy to the results obtained with MSA, [^177^Lu]Lu-6*R*-RedFol-1 and [^177^Lu]Lu-6*S*-RedFol-1 showed slightly stronger binding (97–99%) than [^177^Lu]Lu-OxFol-1 (~ 94%) (Supplementary Material, Fig. S2b). The values of a 50% binding in mouse plasma (B_50_; determined by a semi-log plots) were in the same range for [^177^Lu]Lu-6*R*-RedFol-1 (614 ± 129) and [^177^Lu]Lu-6*S*-RedFol-1 (627 ± 172), but slightly higher for [^177^Lu]Lu-OxFol-1 (747 ± 252) (Supplementary Material, Fig. S2a), resulting in a relative affinity of 1.2 for both [^177^Lu]Lu-6*R*-RedFol-1 and [^177^Lu]Lu-6*S*-RedFol-1, as compared with [^177^Lu]Lu-OxFol-1 which was set as 1.0. The B_50_ values obtained in human plasma were considerably lower resulting in [HSA]-to-[radioconjugate] molar ratios of 149 ± 26 for [^177^Lu]Lu-6*R*-RedFol-1, 184 ± 51 for [^177^Lu]Lu-6*S*-RedFol-1, and 211 ± 39 for [^177^Lu]Lu-OxFol-1 (Supplementary Material, Fig. S2b). This meant relative affinities of 1.4 and 1.2 for [^177^Lu]Lu-6*R*-RedFol-1 and [^177^Lu]Lu-6*S*-RedFol-1, respectively.

### Cell uptake and internalization studies

In vitro studies with KB tumor cells that express the FR revealed a trend of higher uptake of [^177^Lu]Lu-6*R*-RedFol-1 (28–30%) and [^177^Lu]Lu-6*S*-RedFol-1 (42–53%) than for [^177^Lu]Lu-OxFol-1 (20–26%). The internalized fraction of [^177^Lu]Lu-6*R*-RedFol-1 (14–15%) and [^177^Lu]Lu-6*S*-RedFol-1 (14–19%) was also increased as compared with the internalized fraction of [^177^Lu]Lu-OxFol-1 (10–11%). Co-incubation of KB tumor cells with excess of folic acid to block FRs reduced the uptake to < 0.1%, indicating FR-specific uptake of all investigated folate radioconjugates (Supplementary Material, Fig. S3).

### In vivo stability of folate radioconjugates

In vivo stability studies performed in mice without tumors revealed ≥ 99% of intact folate radioconjugates in blood plasma at 4 h after injection of [^177^Lu]Lu-6*R*-RedFol-1, [^177^Lu]Lu-6*S*-RedFol-1, and [^177^Lu]Lu-OxFol-1.

### Biodistribution studies

Biodistribution studies of the folate radioconjugates were performed in KB tumor-bearing mice over a period of 5 days (Table 2; Supplementary Material, Fig. S4, Tables S2/S3). Retention of activity in the blood at 1 h p.i. was higher (*p* < 0.05) for [^177^Lu]Lu-6*R*-RedFol-1 (23 ± 3% IA/g) and [^177^Lu]Lu-6*S*-RedFol-1 (19 ± 2% IA/g) than for [^177^Lu]Lu-OxFol-1 (7.9 ± 1.4% IA/g). At 24 h p.i., blood activity levels were, however, below 3% IA/g in all cases. The uptake of [^177^Lu]Lu-6*R*-RedFol-1 and [^177^Lu]Lu-6*S*-RedFol-1 in KB tumors was significantly increased from 24 h onwards when compared with the uptake of [^177^Lu]Lu-OxFol-1 (*p* < 0.05). The maximum accumulation of [^177^Lu]Lu-6*R*-RedFol-1 in KB tumor xenografts (47 ± 4% IA/g) was reached at 24 h p.i., while in the case of [^177^Lu]Lu-6*S*-RedFol-1, the highest uptake (51 ± 7% IA/g) was observed at 48 h p.i. Both values were much higher than the maximum tumor uptake of [^177^Lu]Lu-OxFol-1 (18 ± 3% IA/g at 24 h p.i.).

**Table 2:** Biodistribution data obtained in KB tumor-bearing mice at 1, 4, 24, 48, 72, and 120 h after injection of [^177^Lu]Lu-6*R*-RedFol-1, [^177^Lu]Lu-6*S*-RedFol-1, and [^177^Lu]Lu-OxFol-1. Data are shown as percentage of the injected activity per gram of tissue [% IA/g], representing the average ± SD

	1 h p.i.	4 h p.i.	24 h p.i.	48 h p.i.	72 h p.i.	120 h p.i.
[^177^Lu]Lu-6*R*-RedFol-1						
Blood	23 ± 3	17 ± 1	5.4 ± 0.9	3.0 ± 0.4	1.0 ± 0.3	0.20 ± 0.13
Kidneys	8.4 ± 0.8	12 ± 1	24 ± 4	18 ± 1	19 ± 5	11 ± 2
Liver	3.3 ± 0.3	3.0 ± 0.2	1.3 ± 0.2	1.1 ± 0.2	0.61 ± 0.14	0.32 ± 0.05
Muscle	1.6 ± 0.2	1.4 ± 0.1	0.82 ± 0.22	0.82 ± 0.06	0.28 ± 0.08	0.28 ± 0.15
Bone	2.1 ± 0.3	1.9 ± 0.2	0.91 ± 0.22	0.75 ± 0.17	0.37 ± 0.09	0.19 ± 0.01
KB tumor	11 ± 1	20 ± 2	47 ± 4	39 ± 5	41 ± 12	23 ± 7
[^177^Lu]Lu-6*S*-RedFol-1						
Blood	19 ± 2	15 ± 2	3.8 ± 0.9	1.0 ± 0.5	0.27 ± 0.11	0.05 ± 0.01
Kidneys	18 ± 2	33 ± 2	69 ± 7	64 ± 6	60 ± 10	28 ± 2
Liver	2.6 ± 0.1	2.3 ± 0.2	0.81 ± 0.22	0.46 ± 0.16	0.28 ± 0.07	0.16 ± 0.03
Muscle	1.6 ± 0.2	1.3 ± 0.1	0.47 ± 0.15	0.21 ± 0.05	0.13 ± 0.06	0.10 ± 0.01
Bone	2.0 ± 0.2	1.7 ± 0.2	0.53 ± 0.08	0.28 ± 0.08	0.16 ± 0.05	0.11 ± 0.01
KB tumor	14 ± 3	26 ± 1	46 ± 3	51 ± 7	42 ± 10	29 ± 3
[^177^Lu]Lu-OxFol-1^1^						
Blood	7.9 ± 1.4	2.3 ± 0.5	0.49 ± 0.08	0.19 ± 0.00	0.10 ± 0.02	0.02 ± 0.01
Kidneys	23 ± 4	22 ± 3	28 ± 3	19 ± 1	17 ± 2	9.5 ± 0.4
Liver	5.0 ± 1.3	3.1 ± 0.4	3.1 ± 1.0	1.9 ± 0.4	1.8 ± 0.6	0.93 ± 0.12
Muscle	1.3 ± 0.1	1.1 ± 0.2	1.0 ± 0.2	0.58 ± 0.26	0.50 ± 0.21	0.19 ± 0.06
Bone	1.6 ± 0.1	1.3 ± 0.2	0.74 ± 0.31	0.50 ± 0.09	0.29 ± 0.09	0.20 ± 0.04
KB tumor	11 ± 1	14 ± 2	18 ± 3	10 ± 1	8.6 ± 0.6	6.0 ± 1.7

^1^Data reproduced with permission from Siwowska et al. 2017, Mol Pharm 14:523 [18]. Copyright 2020 American Chemical Society

[^177^Lu]Lu-6*R*-RedFol-1 and [^177^Lu]Lu-OxFol-1 showed a similar washout after they reached maximum kidney uptake (24 ± 4% IA/g and 28 ± 3% IA/g, respectively) at 24 h. The renal uptake of [^177^Lu]Lu-6*S*-RedFol-1 was much higher (69 ± 7% IA/g; 24 h p.i.). In other tissues such as the liver, lungs, spleen, and heart, the uptake of [^177^Lu]Lu-6*R*-RedFol-1 and [^177^Lu]Lu-6*S*-RedFol-1 was elevated at early timepoints but cleared within 24 h. As a consequence of the distribution profile, the tumor-to-kidney and tumor-to-liver ratios of [^177^Lu]Lu-6*R*-RedFol-1 were significantly increased in comparison with [^177^Lu]Lu-OxFol-1, whereas in the case of [^177^Lu]Lu-6*S*-RedFol-1, only tumor-to-liver ratios were improved (Supplementary Material, Fig. S5).

The uptake of activity in KB tumors and kidneys at 1 h p.i. of all folate radioconjugates was reduced to ~ 5–7% IA/g and ~ 5–9% IA/g, respectively, when folic acid was pre-injected to block FRs in these tissues (Supplementary Material, Table S4).

### Determination of AUC

The AUC_0 → 120h_ of the tumor uptake after injection of [^177^Lu]Lu-6*R*-RedFol-1 and [^177^Lu]Lu-6*S*-RedFol-1 revealed similar values, which were at least 3-fold higher than the respective value of [^177^Lu]Lu-OxFol-1 (Fig. 2; Table 3; Supplementary Material, Table S5) [18]. The AUC_0 → 120h_ of kidney uptake was ~ 3-fold higher for [^177^Lu]Lu-6*S*-RedFol-1 than for [^177^Lu]Lu-6*R*-RedFol-1 and [^177^Lu]Lu-OxFol-1. Consequently, [^177^Lu]Lu-6*R*-RedFol-1 revealed a 3.6-fold increased tumor-to-kidney AUC_0 → 120h_ ratio as compared with [^177^Lu]Lu-OxFol-1, while tumor-to-kidney AUC_0 → 120h_ ratios of [^177^Lu]Lu-6*S*-RedFol-1 and [^177^Lu]Lu-OxFol-1 were similar (Table 3). The tumor-to-blood AUC_0 → 120h_ ratio of [^177^Lu]Lu-6*R*-RedFol-1 was lower than for [^177^Lu]Lu-OxFol-1 while no change in this regard was observed for [^177^Lu]Lu-6*S*-RedFol-1. The tumor-to-liver AUC_0 → 120h_ ratios of [^177^Lu]Lu-6*R*-RedFol-1 and [^177^Lu]Lu-6*S*-RedFol-1 were 6-fold and 11-fold higher as compared with the tumor-to-liver AUC_0 → 120h_ ratio of [^177^Lu]Lu-OxFol-1.

**Table 3:** Areas under the curve up to 120 h p.i. (AUC_0 → 120h_) calculated as [(% IA/g)·h] and tumor-to-background AUC_0 → 120h_ ratios presented as a value relative to [^177^Lu]Lu-OxFol-1 which was set as 1.0

	[^177^Lu]Lu-6*R*-RedFol-1	[^177^Lu]Lu-6*S*-RedFol-1	[^177^Lu]Lu-OxFol-1
Relative AUC_0 → 120h_			
KB tumor	3.2 ± 0.3	3.6 ± 0.3	1.0 ± 0.1
Blood	4.5 ± 0.2	3.3 ± 0.2	1.0 ± 0.1
Kidneys	0.90 ± 0.07	2.8 ± 0.2	1.0 ± 0.1
Liver	0.53 ± 0.02	0.32 ± 0.02	1.0 ± 0.2
Relative AUC_0 → 120h_ ratios			
AUC_Tu_-to-AUC_Bl_	0.71 ± 0.09	1.1 ± 0.2	1.0 ± 0.2
AUC_Tu_-to-AUC_Ki_	3.6 ± 0.6	1.3 ± 0.2	1.0 ± 0.1
AUC_Tu_-to-AUC_Li_	6.1 ± 0.8	11 ± 2	1.0 ± 0.2

### SPECT/CT imaging studies

**Fig. 3:**
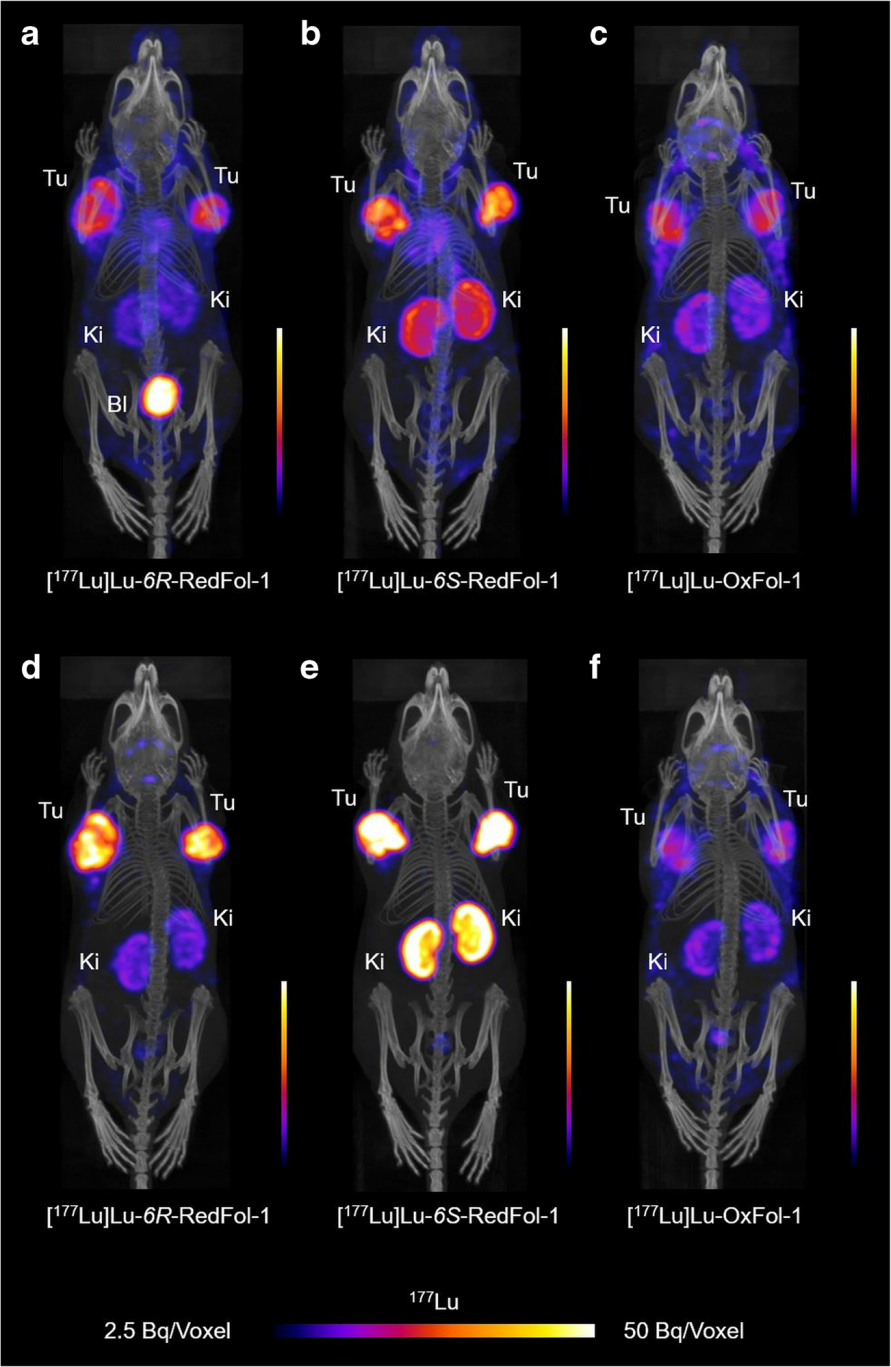
SPECT/CT images shown as maximum intensity projections (MIPs) of KB tumor-bearing mice after injection of the ^177^Lu-folate radioconjugates (25 MBq; 0.5 nmol per mouse). **a**–**c** MIPs obtained at 4 h p.i. **d**–**f** MIPs obtained at 24 h p.i. Tu, KB tumor; Ki, kidney; Bl, urinary bladder

The SPECT/CT images of mice injected with [^177^Lu]Lu-6*R*-RedFol-1 showed high accumulation of activity in the tumors and less in the kidneys as compared with [^177^Lu]Lu-OxFol-1 (Fig. 3). The same high tumor uptake was observed after injection of [^177^Lu]Lu-6*S*-RedFol-1; however, in this case also, the kidney uptake was increased. [^177^Lu]Lu-OxFol-1 demonstrated lower activity in the tumor tissue and a similar kidney retention as observed for [^177^Lu]Lu-6*R*-RedFol-1. The images visualized clearly higher tumor-to-kidney ratios of [^177^Lu]Lu-6*R*-RedFol-1 as compared with [^177^Lu]Lu-6*S*-RedFol-1 and [^177^Lu]Lu-OxFol-1. Pre-injection of mice with folic acid resulted in an almost entire blockade of the tumor and kidney accumulation of all three radioconjugates up to 4 h p.i. In order to block the uptake efficiently also at 24 h p.i., pre-injection of excess albumin-binding folate (cm13) was, however, necessary in particular in the case of [^177^Lu]Lu-6*S*-RedFol-1 (Supplementary Material, Figs. S6–S8).

### Therapy study

**Fig. 4:**
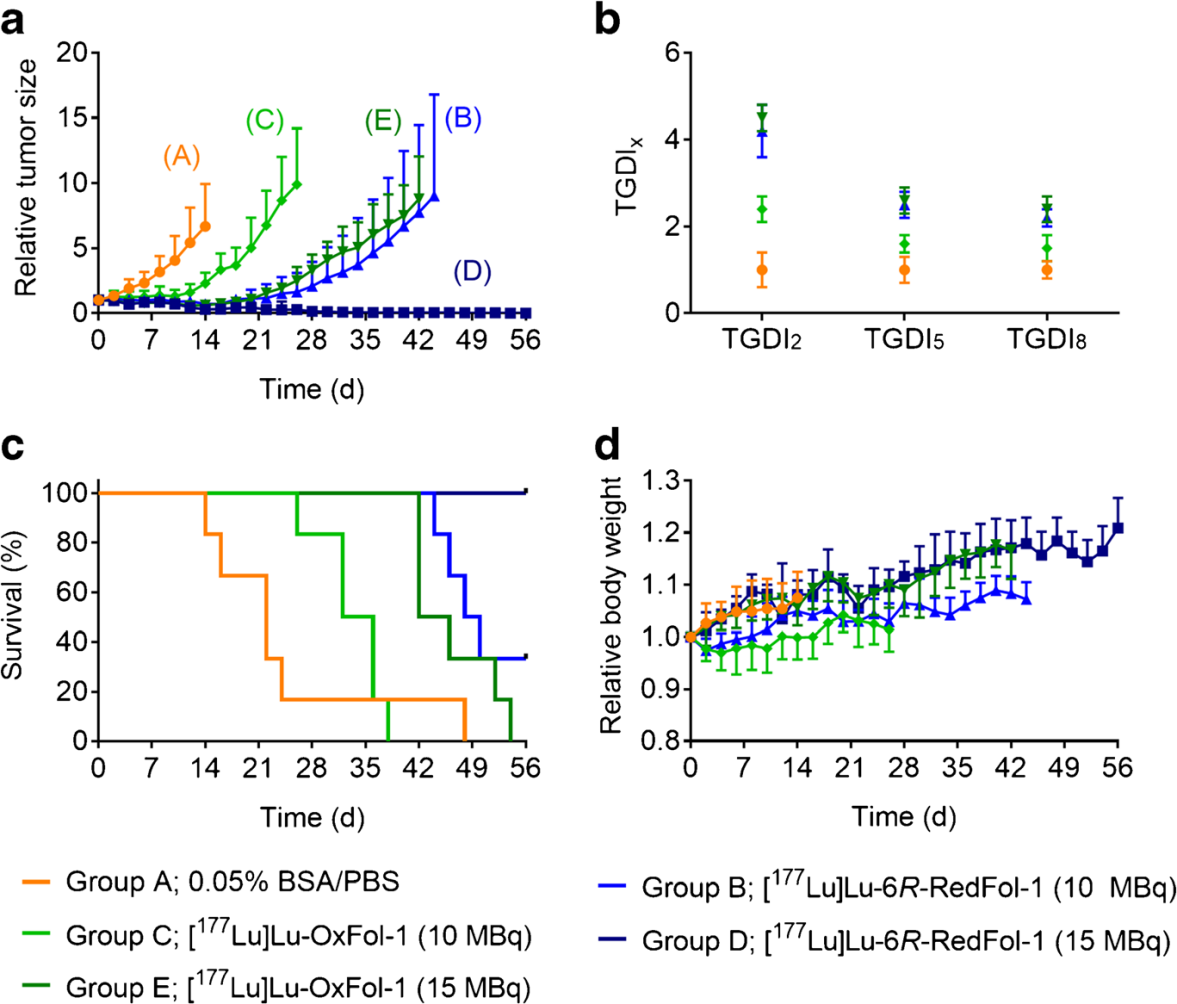
**a** Tumor growth curves relative to the tumor volume at day 0 (set as 1.0) for mice that received PBS (group A), mice treated with [^177^Lu]Lu-6*R*-RedFol-1 (group B/D) or [^177^Lu]Lu-OxFol-1 (group C/E). **b** TGDI_2_, TGDI_5_, and TGDI_8_ determined for respective groups (TGDIs of group D were not determined due to RTV below the threshold value). **c** Kaplan-Meier plot of groups A–E. **d** Relative body weight of groups A–E. Tumor growth and body weights are shown until the first mouse of the respective group reached the endpoint

The tumor size of untreated control mice (group A) was constantly increasing over time, whereas a considerable tumor growth delay was observed in treated mice of groups B–E. This was reflected by significantly increased tumor growth delay indices (TGDI) in treated groups as compared with control mice where the TGDIs were defined as 1.0 (Fig. 4; Supplementary Material, Tables S6/S7).

A significantly more pronounced tumor growth delay was observed in mice treated with 10 MBq [^177^Lu]Lu-6*R*-RedFol-1 (group B) than in the mice treated with 10 MBq [^177^Lu]Lu-OxFol-1 (group C) resulting in increased TGDI_2_ (group B: 4.2 ± 0.6 vs. group C: 2.4 ± 0.3; *p* < 0.05) (Fig. 4a, b; Supplementary Material, Tables S6/S7). Mice that received 15 MBq [^177^Lu]Lu-6*R*-RedFol-1 (group D) showed complete remission of the tumors. Consequently, it was not possible to calculate TGDIs. Interestingly, the mice that received 15 MBq [^177^Lu]Lu-OxFol-1 (group E) had a TGDI_2_ of 4.5 ± 0.3 which was similar to the results observed with 10 MBq [^177^Lu]Lu-6*R*-RedFol-1 (*p* > 0.05). These findings were also reflected by the tumor growth inhibition (TGI) determined at day 14 when the first mouse of control group had to be euthanized (Table 4).

**Table 4:** Data regarding euthanasia period and median survival of mice

Group	Treatment	Time frame of euthanasia (days)	Median survival (days)	TGI (%)
A	0.05% BSA/PBS	14–48	22	0
B	[^177^Lu]Lu-6*R*-RedFol-1 (10 MBq)	44–56*	49	89 ± 3
C	[^177^Lu]Lu-OxFol-1 (10 MBq)	26–38	34	66 ± 12
D	[^177^Lu]Lu-6*R*-RedFol-1 (15 MBq)	56*	n.d.	96 ± 2
E	[^177^Lu]Lu-OxFol-1 (15 MBq)	42–54	44	91 ± 4

n.d. = not determined since all mice survived until the end of the study

*Day 56 = end of the study

The median survival of treated mice was increased compared with the median survival of control mice (group A) (Table 4). The median survival of 49 days for mice treated with 10 MBq [^177^Lu]Lu-6*R*-RedFol-1 (group B) was much longer than for mice that received 10 MBq [^177^Lu]Lu-OxFol-1 (group C: 34 days). All mice injected with 15 MBq [^177^Lu]Lu-6*R*-RedFol-1 (group D) survived until the end of the study at day 56, whereas mice that received 15 MBq [^177^Lu]Lu-OxFol-1 (group E) had a median survival of only 44 days.

### Assessment of the therapy study

The average relative body weight (1.00–1.08) was comparable in all groups of mice at day 14, when the first mouse of the control group reached an endpoint (Supplementary Material, Table S8). Organ-to-body weight as well as organ-to-brain mass ratios may serve as indicators of the health status, since it is known that the brain of the mice does not increase in size after the age of 3 weeks (Supplementary Material, Tables S9/S10) [25, 26]. The calculated organ-to-brain mass ratios were in the same range for untreated mice and mice treated with 10 MBq of the folate radioconjugates (groups B/C). The organ-to-brain mass ratios of mice treated with 15 MBq of the folate radioconjugates (groups D/E) were decreased (*p* < 0.05).

Blood plasma parameters determined at the time of euthanasia did not differ among treated mice and untreated controls (Supplementary Material, Table S11). Histological investigations of the kidneys, spleen, and bone marrow did, however, not reveal any significant lesion attributed to the treatment. In particular, bone marrow of mice that received [^177^Lu]Lu-6*R*-RedFol-1 showed an overall hematopoietic cellularity comparable to the control animals and mice treated with [^177^Lu]Lu-OxFol-1 (Supplementary Material, Table S12).

## Discussion

In this study, albumin-binding radioconjugates of a new class, based on 5-MTHF as a FR-binding entity, were evaluated and compared with the previously developed [^177^Lu]Lu-OxFol-1 [18]. [^177^Lu]Lu-6*R*-RedFol-1 and [^177^Lu]Lu-6*S*-RedFol-1 showed high stability in PBS and human plasma in vitro. In all three cases, the binding to human plasma proteins was stronger than to mouse plasma proteins, which is in line with the reported affinity of the *p*-iodophenyl entity [27] and recently reported results obtained with albumin-binding PSMA-targeted radioligands [23]. In vitro studies revealed that the exchange of folic acid with 5-MTHF slightly increased the affinity of the respective radioconjugates to both mouse and human plasma proteins when compared with the affinity determined for [^177^Lu]Lu-OxFol-1. It remains, however, unclear whether this was the reason for the increased blood retention of 5-MTHF-based folate radioconjugates or if it was due to another, yet unknown, mechanism. The hypothesis that the observed phenomenon was due to radiometabolite formation was refuted by stability experiments that showed only intact folate radioconjugates in the blood plasma of mice 4 h after injection. The FR-specific in vitro uptake of the radioconjugates into KB tumor cells was demonstrated in vivo, since / in vivo, as pre-injection of excess folic acid reduced the ac-cumulation of the radioconjugates in tumors and kidneys of mice.The application of excess non-labeled albumin-binding folic acid conjugate (cm13) was, however, much more effective in this regard due to its enhanced blood circulation similar to the folate radioconjugates in question.

[^177^Lu]Lu-6*R*-RedFol-1 and [^177^Lu]Lu-6*S*-RedFol-1 showed a somewhat higher in vitro uptake and internalization in FR-positive KB tumor cells as compared with [^177^Lu]Lu-OxFol-1, and the in vivo results revealed even a 3–4-fold increased tumor uptake of [^177^Lu]Lu-6*R*-RedFol-1 and [^177^Lu]Lu-6*S*-RedFol-1. The in vivo results were likely due to the enhanced blood retention of the 5-MTHF-based radioconjugates; however, it may also be due to the easier release of 5-MTHF from the FR upon internalization and, thus, more efficient accumulation as compared with folic acid [28]. The latter hypothesis is further supported by the results of Boss et al. who observed an increased tumor uptake of ^18^F-labeled 5-MTHF radiotracers as compared with the folic acid analogue even though these radiotracers did not comprise an albumin-binding entity [21]. In parallel to the increased tumor uptake, [^177^Lu]Lu-6*S*-RedFol-1 showed also higher retention in the kidneys resulting in similar tumor-to-kidney ratios as observed with [^177^Lu]Lu-OxFol-1. In the case of [^177^Lu]Lu-6*R*-RedFol-1, the retention in the kidneys remained relatively low resulting in substantially improved tumor-to-kidneys AUC_0 → 120h_ ratios to a value that has never been achieved before. This radioconjugate was, thus, selected for further investigations in a preclinical therapy study.

As expected, the treatment of KB tumor-bearing mice with 10 MBq or 15 MBq [^177^Lu]Lu-6*R*-RedFol-1, showed an activity-dependent tumor growth inhibition and survival of mice. In line with the higher tumor uptake, the outcome of the [^177^Lu]Lu-6*R*-RedFol-1 therapy was superior over that of [^177^Lu]Lu-OxFol-1. Comparison of the TGDI and TGI results suggested that the application of 10 MBq [^177^Lu]Lu-6*R*-RedFol-1 was equipotent to the application of 15 MBq [^177^Lu]Lu-OxFol-1 which confirmed the enhanced therapeutic potential of this novel radioconjugate.

As the tumor-to-kidney AUC_0 → 120h_ ratio of [^177^Lu]Lu-6*R*-RedFol-1 was almost 4-fold increased compared with [^177^Lu]Lu-OxFol-1, the use of [^177^Lu]Lu-6*R*-RedFol-1 would most probably allow delivering an effective tumor dose without the risk of long-term damage to the kidneys. In our study, no obvious early side effects were observed. Neither the body weights nor the blood plasma parameters of treated mice were significantly different from the control values. Moreover, no significant histopathological changes in kidneys and spleen were observed that would indicate radiation-induced damage of these tissues. Most importantly, the evaluation of the bone marrow of mice treated with [^177^Lu]Lu-6*R*-RedFol-1 confirmed the absence of hematological side effects, in spite of the enhanced blood retention of this novel radioconjugate.

## Conclusion

It was demonstrated in this study that 5-MTHF-based radioconjugates have the potential to be used for targeted radionuclide therapy. Due to the unprecedentedly high tumor-to-kidney ratios of [^177^Lu]Lu-6*R*-RedFol-1, this radioconjugate outperformed any other folate radioconjugate. It was, thus, shown to have enhanced therapeutic efficacy as compared with [^177^Lu]Lu-OxFol-1, which makes [^177^Lu]Lu-6*R*-RedFol-1 attractive for clinical translation.

## Electronic supplementary material

ESM 1 comprises information about: the radiolabeling of the folate conjugates, in vitro studies, methods of imaging and biodistribution data, including determination of AUC values and additional information regarding the therapy assessment and results. (PDF 9861 kb)

## Abbreviations

AUC: area under the curve
BSA: bovine serum albumin
BW: body weight
cpm: counts per minute
CT: computed tomography
DOTA: 1,4,7,10-tetraazacyclododecane-1,4,7,10-tetraacetic acid
FR: folate receptor
FWHM: full width at half maximum
HPLC: high-performance liquid chromatography
HSA: human serum albumin
IA: injected activity
MSA: mouse serum albumin
5-MTHF: 5-methyltetrahydrofolate
n.d.: not determined
PBS: phosphate-buffered saline
PET: positron emission tomography
p.i.: post-injection
PSMA: prostate-specific membrane antigen
RBW: relative body weight
RPMI: Roswell Park Memorial Institute
RTV: relative tumor volume
SD: standard deviation
SPECT: single-photon emission computed tomography
TGD: tumor growth delay
TGI: tumor growth inhibition
TGDI: tumor growth delay index
TV: tumor volume
