# Supplementary material for: Preclinical evaluation of 5-methyltetrahydrofolate-based radioconjugates—new perspectives for folate receptor–targeted radionuclide therapy
Source: Eur J Nucl Med Mol Imaging. 2020 Oct 15;48(4):972–83. doi: 10.1007/s00259-020-04980-y (PMC8041685; doi:10.1007/s00259-020-04980-y)
Supplement: Supplementary file 1 — comprises information about: the radiolabeling of the folate conjugates, in vitro studies, methods of imaging and biodistribution data, including determination of AUC values and additional information regarding the therapy assessment and results. (PDF 9861 kb) [file 259_2020_4980_MOESM1_ESM.pdf]

SUPPLEMENTARY MATERIAL

Preclinical evaluation of 5-methyltetrahydrofolate-based radioconjugates – new perspectives for folate receptor-targeted radionuclide therapy

Patrycja Guzik[1], Martina Benešová[1,2], Magdalena Ratz[1], Josep M. Monné Rodríguez[3], Luisa M. Deberle[2], Roger Schibli[1,2], Cristina Müller*[,1,2]

[1] *Center for Radiopharmaceutical Sciences ETH-PSI-USZ, Paul Scherrer Institute, Villigen-PSI, Switzerland*

[2] *Department of Chemistry and Applied Biosciences, ETH Zurich, Zurich, Switzerland*

[3] *Laboratory for Animal Model Pathology (LAMP), Institute of Veterinary Pathology, Vetsuisse Faculty, University of Zurich, Zurich, Switzerland*

E-Mail addresses:
patrycja.guzik@psi.ch; martina.benesova@t-online.de; magdalena.ratz@gmx.at; josep.monnerodriguez@uzh.ch; luisa.deberle@psi.ch; roger.schibli@psi.ch; cristina.mueller@psi.ch

*** Correspondence to:**
PD Dr. Cristina Müller
Center for Radiopharmaceutical Sciences ETH-PSI-USZ
Paul Scherrer Institute
5232 Villigen-PSI
Switzerland
e-mail: cristina.mueller@psi.ch
phone: +41-56-310 44 54
fax: +41-56-310 28 49

1. Radiolabeling and quality control of folate radioconjugates

Purpose: Folate conjugates were labeled with lutetium-177 for in vitro and in vivo experiments.

Methods: Lutetium-177 was added to a mixture of HCl (0.05 M) and Na-acetate (0.5 M, pH 8) containing the respective folate conjugate to obtain a molar activity of 10-50 MBq/nmol. L-Ascorbic acid (6 mg) was added to prevent oxidation. The reaction mixture was incubated at 95 °C for 15 min. The radiolabeling of OxFol-1 was performed as previously reported without addition of L-ascorbic acid [1]. Quality control of the radiolabeled folate conjugates, diluted in MilliQ water containing sodium diethylenetriamine pentaacetic acid (Na-DTPA; 50 µM), was performed with a Merck Hitachi LaChrom HPLC system, equipped with a D-7000 interface, a L-7200 autosampler, a radiation detector (LB 506 B from Berthold) and a L-7100 pump connected with a reversed-phase C18 column (5 µm, 150×4.6 mm, Xterra™, MS, Waters, U.S.A). The mobile phase consisted of 0.1% trifluoroacetic acid in MilliQ water (A) and acetonitrile (B) using a linear gradient of solvent A (95–20% over 15 min) in solvent B at a flow rate of 1 mL/min.

Results: The HPLC retention times were equal for all three folate radioconjugates (Fig. S1). The radiofolates were used for in vitro and in vivo experiments without further purification.

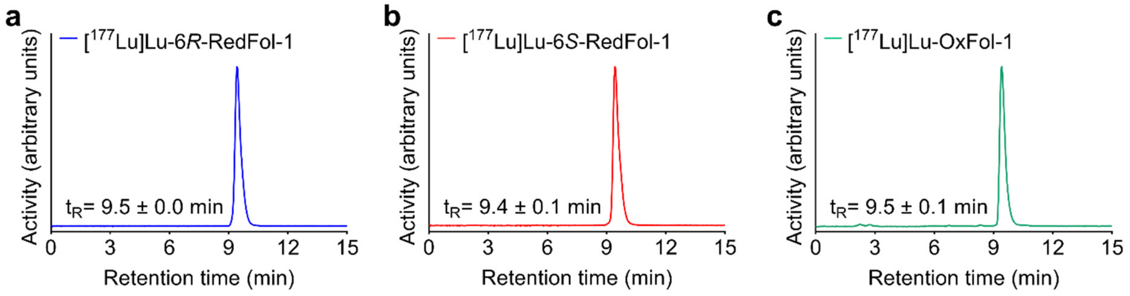

Fig. S1 Representative chromatograms obtained after radiolabeling of 6*R*-RedFol-1, 6*S*-RedFol-1 and OxFol-1 with lutetium-177. (**a**) [^{177}Lu]Lu-6*R*-RedFol-1; (**b**) [^{177}Lu]Lu-6*S*-RedFol-1; (**c**) [^{177}Lu]Lu-OxFol-1. (Retention times (t_R) are indicated as the average ± standard deviation (SD), n = 6). The presence of uncoordinated lutetium-177 detected as [^{177}Lu]Lu-DTPA would appear with t_R = 2.4 ± 0.2 min.

Remark: Throughout all studies, the indicated molar activities refer to the time of radioconjugate preparation. Due to the significantly longer half-life of lutetium-177 ($T_{1/2}$ = 6.65 d) compared to the time needed for the preparation and quality control of the radioconjugates (~1 h), the decay was neglected.

2. Stability of the folate radioconjugates

Purpose: The stability of the folate radioconjugates was determined to assess the period in which they could be used for experiments.

Methods: After the quality control was performed using HPLC (t = 0), the labeling solutions of [^{177}Lu]Lu-6*R*-RedFol-1 and [^{177}Lu]Lu-6*S*-RedFol-1 (50 MBq/nmol) were diluted with PBS to obtain an activity concentration of 100 MBq/500 µL. Sodium acetate (130 µL, 0.5 M, pH 8) was added to the sample to maintain a neutral pH value. The integrity of the folate radioconjugates was determined by HPLC over a period of 24 h. The HPLC chromatograms were analyzed by determination of the peak area of the radiolabeled product, the released lutetium-177 as well as degradation products of unknown structure. The quantity of the intact product was expressed as percentage of the sum of integrated peak areas of the entire chromatogram and set into relation to the original value determined at t = 0, which was set as 100%.

Results: The results of the stability assessment of [^{177}Lu]Lu-6*R*-RedFol-1 and [^{177}Lu]Lu-6*S*-RedFol-1 were in the same range as those previously reported for [^{177}Lu]Lu-OxFol-1 when tested under the same conditions (Table S1).

Table S1 Stability of folate radioconjugates determined after variable incubation periods, expressed as percentage of the sum of integrated peak areas of the entire chromatogram, which was set to 100%

Radioconjugate	Activity Concentration	Intact radiolabeled product (%)			
		1 h	2 h	4 h	24 h
[^{177}Lu]Lu-6*R*-RedFol-1	100 MBq/500 µL	≥99	≥99	≥99	≥97
[^{177}Lu]Lu-6*S*-RedFol-1	100 MBq/500 µL	≥99	≥99	≥99	≥97
[^{177}Lu]Lu-OxFol-1[1]	100 MBq/500 µL	n.d.	n.d.	≥99	≥97

[1] Data for [^{177}Lu]Lu-OxFol-1 were previously published by Siwowska et al. 2017 [1]

n.d. = not determined

3. In vitro stability of folate radioconjugates in human plasma

Purpose: Investigation of plasma stability was performed for quantitative analysis of intact folate radioconjugates and potential radiometabolites formed in the plasma.

Methods: Radiolabeling of folate conjugates was performed according to the same procedure as described for the stability experiments in PBS. Quality control of the radiolabeled folate conjugates was performed as described above, however, the HPLC program was adapted. The mobile phase consisted of 0.1% trifluoroacetic acid in MilliQ water (A) and acetonitrile (B) using a linear gradient of solvent A (95–50%) and solvent B (5–50%) over 35 min at a flow rate of 0.8 mL/min. After dilution of radioconjugates to 30 MBq/500 µL in PBS 7.4, a sample of 50 µL was mixed with 250 µL human plasma (Stiftung Blutspende SRK Aargau-Solothurn, Switzerland) and incubated at 37 °C. After 4 h and 24 h

of incubation, a sample of 50 µL was mixed with 150 µL cold methanol containing ammonium hydroxide (0.025%) and 2-mercaptoethanol (10 mg/mL) for precipitation of the proteins. After centrifugation (4 °C, 3 min, 8000 rpm), the supernatant was collected and centrifuged for a second time. After filtration of the supernatant through a polytetrafluoroethylene membrane filter (0.2 µm), the samples were injected into HPLC. The chromatograms were analyzed by determination of the peak area of the radiolabeled product, the released lutetium-177 as well as degradation products of unknown structure. The quantity of the intact product was expressed as percentage of the sum of integrated peak areas of the entire chromatogram and set into relation to the original value determined for the quality control, which was set as 100%.

Results: The results are reported in the main article.

4. Determination of logD values

Purpose: The distribution coefficients (logD values) of the folate radioconjugates were determined for comparison.

Methods: The folate conjugates were labeled at a molar activity of 50 MBq/nmol. A sample of each folate radioconjugate (~0.5 MBq, 25 µL) was mixed with 1475 µL PBS pH 7.4 and 1500 µL *n*-octanol. The vials were vortexed vigorously for 1 min and then centrifuged (2500 rpm) for 6 min for phase separation. The activity concentration in a defined volume of each layer was measured in a γ-counter (Perkin Elmer, Wallac Wizard 1480). The distribution coefficients were calculated as the logarithm of the ratio of counts per minute (cpm) measured in the *n*-octanol phase relative to the cpm measured in the PBS pH 7.4 phase. The results were reported as mean ± standard deviation (SD) of the data obtained from three to five independent experiments, each performed with five replicates.

Results: The results are reported in the main article.

5. Binding affinity to mouse and human plasma proteins

Purpose: The aim of this experiment was to investigate and compare the albumin-binding properties of the folate radioconjugates.

Methods: The percentage of bound folate radioconjugate to mouse and human serum albumin was determined at different albumin-to-radioligand molar ratios, by means of an ultrafiltration assay [2]. The amount of albumin in mouse plasma (Rockland, USA) and human plasma (Stiftung Blutspende SRK Aargau-Solothurn, Switzerland) was determined using a dry chemistry analyzer (DRI-CHEM 4000i, FUJIFILM, Japan) and was found to be 550 µM and 850 µM, respectively. An aliquot of the diluted radioconjugate (~500 kBq, 25 µL, 0.01 nmol) was mixed with diluted plasma in molar ratios of mouse serum albumin (MSA)-to-folate conjugate or human serum album (HSA)-to-folate conjugate in the range of 0.01–21250. The highest molar ratio represented the situation of the radioligand in undiluted plasma. Afterwards, samples were incubated for 30 min at 37 °C. Aliquots of the samples were loaded

to the ultrafiltration device (Centrifree ultrafiltration devices; 30000 Da nominal molecular weight limit, methyl-cellulose micropartition membranes, Millipore) and centrifuged at 800 rcf for 40 min at 20 °C. The activity in the filtrate as well as aliquots of the incubation solutions were measured in a γ-counter (Perkin Elmer, Wallac Wizard 1480). The albumin-bound fraction was calculated based on the measurement of the filtrates, which corresponded to the free fractions, and was expressed as the percentage of total added activity, which was set to 100%. In a control experiment, the ^{177}Lu-folate conjugates were filtered after incubation in PBS instead of plasma. In this case, the majority of activity (>90%) was found in the filtered fraction indicating that the unbound folate radioconjugates were readily filtered through the membrane. The results were presented as average ± SD of three independent experiments. The data were fitted to a semi-logarithmic plot using non-linear regression (one-site, specific binding) to obtain the half maximum binding (B_{50}) using GraphPad Prism software (version 7). The B_{50} value of [^{177}Lu]Lu-OxFol-1 was set to 1.0. The inverse ratio of the B_{50} of [^{177}Lu]Lu-6*R*-RedFol-1 and [^{177}Lu]Lu-6*S*-RedFol-1 was calculated to allow the comparison of the relative binding affinity of each radioconjugate.

Results: The results are reported in the main article and visualized below (Fig. S2).

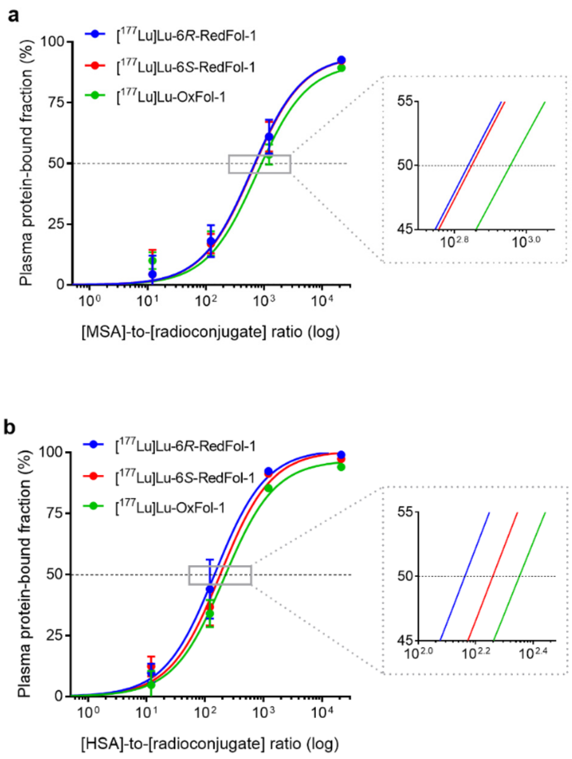

Fig. S2 Plasma protein-binding curves of the folate radioconjugates. (**a**) Affinity curves obtained in mouse plasma; (**b**) Affinity curves obtained in human plasma. The plasma was diluted to defined molar ratios of mouse or human albumin-to-folate radioconjugate. (MSA = mouse serum albumin; HSA = human serum albumin).

6. Cell uptake and internalization studies

Purpose: The purpose of this experiment was to compare the in vitro properties the folate radioconjugates with regard to the uptake and internalization into FR-positive KB tumor cells.

Methods: KB tumor cells were seeded in 12-well plates (0.5×10^6 cells in 2 mL folate free RPMI (FFRPMI) medium/well) allowing cell adhesion and growth overnight at 37 °C and 5% CO_2. After removal of the supernatant, cells were washed with PBS prior to the addition of FFRPMI medium without supplements (975 µL/well). The ^{177}Lu-folate conjugates (50 MBq/nmol) were added to each well in a volume of 25 µL (0.75 pmol, 38 kBq). In some wells, tumor cells were co-incubated with excess folic acid (100 µM) to block FRs on the cell surface. After incubation of the well plates for 2 h or 4 h at 37 °C and 5% CO_2, cells were washed three times with ice-cold PBS to determine total uptake of the folate radioconjugates. In order to assess the internalized fraction, a stripping buffer (aqueous solution of 0.1 M acetic acid and 0.15 M NaCl, pH 3) was applied to release FR-bound folate radioconjugates from the cell surface. Cell samples were lysed by addition of NaOH (1 M, 1 mL) to each well. The radioactive cell lysates were measured in a γ-counter (Perkin Elmer, Wallac Wizard 1480). After homogenization of the cell suspensions by vortexing, the concentration of proteins was determined for each sample by a Micro BCA Protein Assay kit (Pierce, Thermo Scientific) in order to standardize measured activity to the average content of protein (~0.2 mg) in a single well. The results were expressed as percentage of total added activity.

Results: Compared to [^{177}Lu]Lu-OxFol-1, both [^{177}Lu]Lu-RedFol-1 conjugates showed higher uptake in the KB tumor cells (Fig. S3a). [^{177}Lu]Lu-6*S*-RedFol-1 showed the highest uptake in KB tumor cells, which was in the range of 42% and 53% after 2 h and 4 h, respectively.

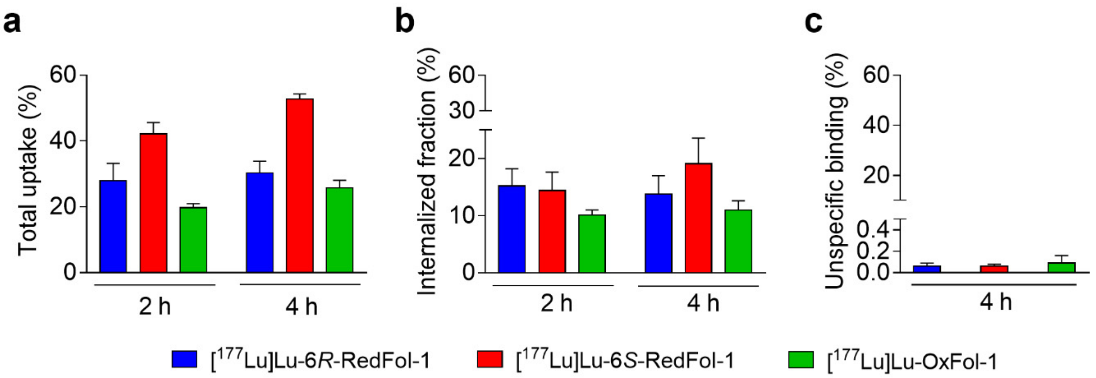

Fig. S3 Results of the in vitro tumor cell uptake of [^{177}Lu]Lu-6*R*-RedFol-1, [^{177}Lu]Lu-6*S*-RedFol-1 and [^{177}Lu]Lu-OxFol-1 in KB tumor cells after 2 h and 4 h of incubation at 37 ºC. (**a**) Tumor cell uptake; (**b**) Internalized fraction; (**c**) Unspecific binding.

The uptake of [^{177}Lu]Lu-6*R*-RedFol-1 was lower and in the range of 30% at both investigated time points. The internalized fraction was in the range of 10-19% for all three folate radioconjugates and did

not change significantly during the 4 h-incubation period (Fig. S3b). Co-incubation of the tumor cells with excess folic acid to block the FRs reduced the uptake to <0.1%, indicating FR-specific uptake of all investigated folate radioconjugates (Fig. S3c).

7. In vivo stability of folate radioconjugates in blood plasma

Purpose: Investigation of plasma stability was performed for determination of intact folate radioconjugates and potential radiometabolites formed in the blood plasma of mice.

Methods: Radiolabeling of folate conjugates and quality control were performed according to the same procedure as for the in vitro stability experiments. [^{177}Lu]Lu-6*R*-RedFol-1, [^{177}Lu]Lu-6*S*-RedFol-1 and [^{177}Lu]Lu-OxFol-1 were diluted in PBS pH 7.4 containing 0.05% BSA and injected into CD-1 Foxn-1/nu mice (Charles River) without tumors (25 MBq, 0.5 nmol, 100 µL). Blood was drawn from the retrobulbar vein under anesthesia 4 h after injection of the respective folate radioconjugate followed by immediate euthanasia of the mouse. Blood samples were centrifuged (4 °C, 20 min, 1600 rpm) to allow collecting blood plasma. A volume of 450 µL cold methanol containing ammonium hydroxide (0.025%) and 2-mercaptoethanol (10 mg/mL) was added to 150 µL plasma for precipitation of the proteins. After centrifugation (4 °C, 3 min, 8000 rpm), the supernatant was collected and centrifuged for a second time. After filtration of the supernatant through a polytetrafluoroethylene membrane filter (0.2 µm) the samples were injected into HPLC and the chromatograms analyzed in the same way as described for in vitro stability experiments.

Results: The results of the in vivo plasma stability of the folate radioconjugates are reported in the main article.

8. Biodistribution studies

Purpose: Biodistribution studies were performed in KB tumor-bearing mice in order to compare the tissue distribution profiles of the novel radioconjugates with that of the previously investigated [^{177}Lu]Lu-OxFol-1 [1]. Moreover, non-decay-corrected biodistribution data points were necessary to calculate the areas under the curve (AUC) values.

Methods: The preparation of the tumor-bearing mice and the experimental setting of the biodistribution studies in tumor-bearing mice are described in the main article. Blocking studies were performed by pre-injecting the mice with folic acid (100 µg/100 µL PBS pH 7.4) ~5 min prior to the respective folate radioconjugate (3 MBq, 0.5 nmol, 100 µL) diluted in PBS containing 0.05% BSA. Mice were sacrificed 1 h after administration of folate radioconjugate. Activity uptake in the collected organs and tissues was quantified in the same way as described in the main article for biodistribution. Biodistribution data were analyzed for significance using a two-way ANOVA test with a Tukey's multiple comparisons post-test (GraphPad Prism software, version 7). A p-value of <0.05 was considered as statistically significant.

Results: The biodistribution data of each folate radioconjugate are discussed in the main article and below (Fig. S4/S5, Tables S2–S4).

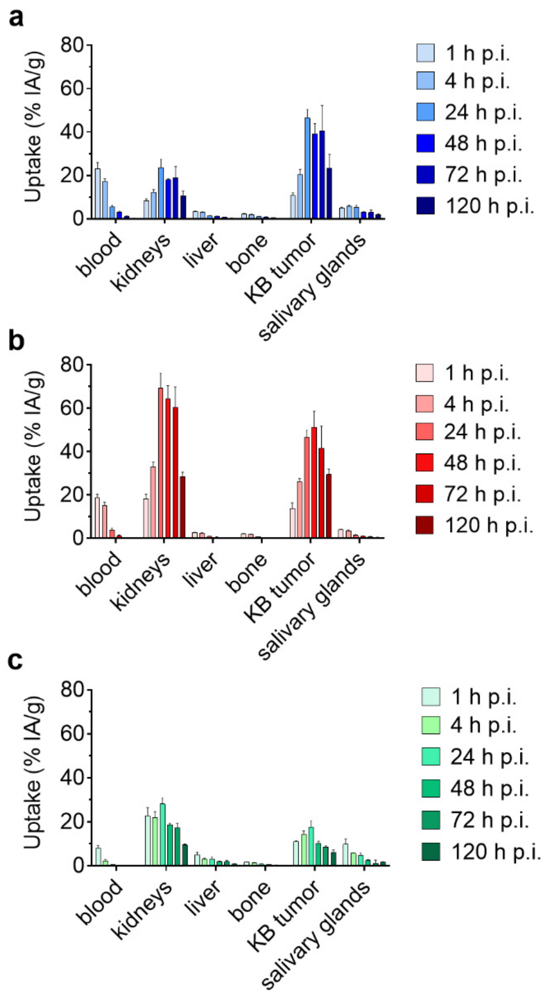

Fig. S4 Graphs representing the decay-corrected tissue distribution profile of the folate radioconjugates over 120 h. (**a**) [^{177}Lu]Lu-6*R*-RedFol-1; (**b**) [^{177}Lu]Lu-6*S*-RedFol-1 and (**c**) [^{177}Lu]Lu-OxFol-1. The data of [^{177}Lu]Lu-OxFol-1 were reproduced from Siwowska et al. [1].

Table S2 Biodistribution data and tumor-to-background ratios obtained in KB tumor-bearing mice at various time points after injection of [^{177}Lu]Lu-6*R*-RedFol-1. Decay-corrected data of accumulated activity are shown as % IA/g tissue, representing the average ± SD.

**Organs**	**[^{177}Lu]Lu-6*R*-RedFol-1**					
	**1 h p.i.**	**4 h p.i.**	**24 h p.i.**	**48 h p.i.**	**72 h p.i.**	**120 h p.i.**
Blood	23 ± 3	17 ± 1	5.4 ± 0.9	3.0 ± 0.4	1.0 ± 0.3	0.20 ± 0.13
Lung	10 ± 1	7.9 ± 1.4	3.2 ± 0.6	2.4 ± 0.2	0.99 ± 0.24	0.37 ± 0.08
Spleen	2.6 ± 0.2	2.2 ± 0.2	1.7 ± 0.1	1.5 ± 0.1	1.3 ± 0.4	0.95 ± 0.14
Kidneys	8.4 ± 0.8	12 ± 1	24 ± 4	18 ± 1	19 ± 5	11 ± 2
Stomach	1.9 ± 0.4	1.9 ± 0.3	0.80 ± 0.11	0.46 ± 0.07	0.29 ± 0.10	0.13 ± 0.02
Intestines	2.9 ± 0.3	2.6 ± 0.7	0.80 ± 0.09	0.53 ± 0.07	0.30 ± 0.09	0.32 ± 0.23
Liver	3.3 ± 0.3	3.0 ± 0.2	1.3 ± 0.2	1.1 ± 0.2	0.61 ± 0.14	0.32 ± 0.05
Salivary gl.	5.0 ± 0.3	5.7 ± 0.5	5.2 ± 1.0	3.0 ± 0.3	3.0 ± 1.1	2.0 ± 0.3
Muscle	1.6 ± 0.2	1.4 ± 0.1	0.82 ± 0.22	0.82 ± 0.06	0.28 ± 0.08	0.28 ± 0.15
Bone	2.1 ± 0.3	1.9 ± 0.2	0.91 ± 0.22	0.75 ± 0.17	0.37 ± 0.09	0.19 ± 0.01
KB Tumor	11 ± 1	20 ± 2	47 ± 4	39 ± 5	41 ± 12	23 ± 7
**Organ Ratios**	**Tumor-to-Background Ratios**					
	**1 h p.i.**	**4 h p.i.**	**24 h p.i.**	**48 h p.i.**	**72 h p.i.**	**120 h p.i.**
Tu-to-blood	0.46 ± 0.01	1.2 ± 0.1	8.8 ± 0.8	13 ± 4	39 ± 8	142 ± 75*
Tu-to-liver	3.3 ± 0.2	6.9 ± 0.7	35 ± 4	37 ± 9*	68 ± 19*	74 ± 12*
Tu-to-kidney	1.3 ± 0.1*	1.7 ± 0.1*	2.0 ± 0.4*	2.2 ± 0.3*	2.1 ± 0.1*	2.2 ± 0.5*

* Significantly different (p<0.05) from the value of [^{177}Lu]Lu-OxFol-1 [1]

Table S3 Biodistribution data and tumor-to-background ratios obtained in KB tumor-bearing mice at various time points after injection of [^{177}Lu]Lu-6*S*-RedFol-1. Decay-corrected data of accumulated activity are shown as % IA/g tissue, representing the average ± SD.

**Organs**	**[^{177}Lu]Lu-6*S*-RedFol-1**					
	**1 h p.i.**	**4 h p.i.**	**24 h p.i.**	**48 h p.i.**	**72 h p.i.**	**120 h p.i.**
Blood	19 ± 2	15 ± 2	3.8 ± 0.9	1.0 ± 0.5	0.27 ± 0.11	0.05 ± 0.01
Lung	8.5 ± 0.6	6.7 ± 0.6	2.2 ± 0.4	0.85 ± 0.31	0.36 ± 0.10	0.15 ± 0.03
Spleen	2.0 ± 0.2	1.9 ± 0.3	1.1 ± 0.2	1.1 ± 0.3	0.89 ± 0.14	0.67 ± 0.11
Kidneys	18 ± 2	33 ± 2	69 ± 7	64 ± 6	60 ± 10	28 ± 2
Stomach	1.5 ± 0.3	1.2 ± 0.2	0.51 ± 0.14	0.23 ± 0.06	0.11 ± 0.03	0.06 ± 0.02
Intestines	1.8 ± 0.4	1.9 ± 0.1	0.49 ± 0.04	0.21 ± 0.09	0.11 ± 0.04	0.06 ± 0.02
Liver	2.6 ± 0.1	2.3 ± 0.2	0.81 ± 0.22	0.46 ± 0.16	0.28 ± 0.07	0.16 ± 0.03
Salivary gl.	4.0 ± 0.2	3.5 ± 0.4	1.4 ± 0.2	0.84 ± 0.23	0.62 ± 0.18	0.45 ± 0.05
Muscle	1.6 ± 0.2	1.3 ± 0.1	0.47 ± 0.15	0.21 ± 0.05	0.13 ± 0.06	0.10 ± 0.01
Bone	2.0 ± 0.2	1.7 ± 0.2	0.53 ± 0.08	0.28 ± 0.08	0.16 ± 0.05	0.11 ± 0.01
KB Tumor	14 ± 3	26 ± 1	46 ± 3	51 ± 7	42 ± 10	29 ± 3
**Organ Ratios**	**Tumor-to-Background Ratios**					
	**1 h p.i.**	**4 h p.i.**	**24 h p.i.**	**48 h p.i.**	**72 h p.i.**	**120 h p.i.**
Tu-to-blood	0.73 ± 0.12	1.7 ± 0.2	13 ± 3	58 ± 23	138 ± 12	580 ± 127*
Tu-to-liver	5.3 ± 1.0	11 ± 1	59 ± 13*	120 ± 29*	152 ± 43*	189 ± 31*
Tu-to-kidney	0.75 ± 0.09	0.79 ± 0.07	0.67 ± 0.08	0.80 ± 0.06	0.68 ± 0.10	1.0 ± 0.1*

* Significantly different (p<0.05) from the value of [^{177}Lu]Lu-OxFol-1 [1]

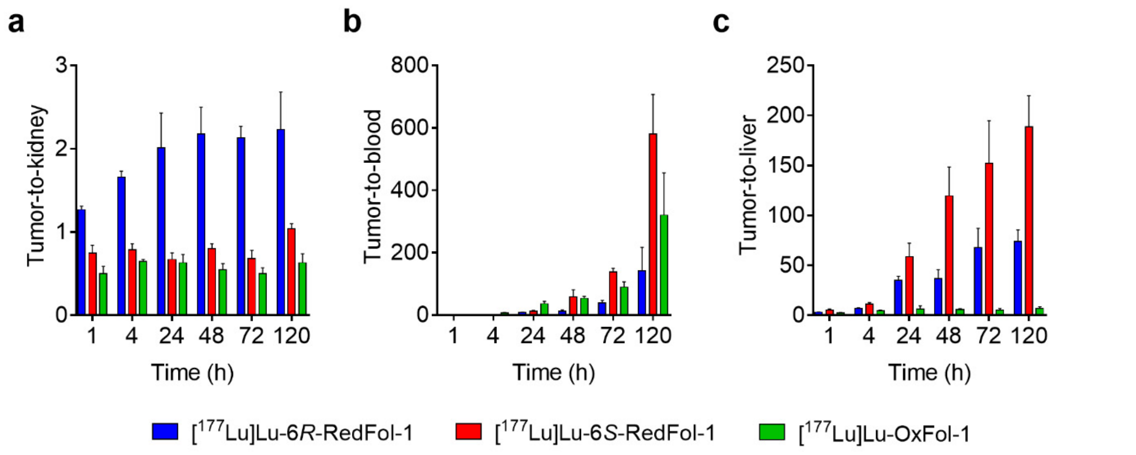

Fig. S5 Comparison of tumor-to-background ratios determined based on biodistribution data obtained at various time points after administration of [^{177}Lu]Lu-6*R*-RedFol-1, [^{177}Lu]Lu-6*S*-RedFol-1 and [^{177}Lu]Lu-OxFol-1: (**a**) Tumor-to-kidney ratios; (**b**) Tumor-to-blood ratios; (**c**) Tumor-to-liver ratios.

The data obtained in mice pre-injected with folic acid are discussed in the main manuscript and listed for each folate radioconjugate below (Table S4).

Table S4 Accumulation of [^{177}Lu]Lu-6*R*-RedFol-1, [^{177}Lu]Lu-6*S*-RedFol-1 and [^{177}Lu]Lu-OxFol-1 in selected organs of KB tumor-bearing mice at 1 h after injection in comparison to the uptake upon FR-blockade with pre-injected folic acid (FA). Decay-corrected data of accumulated activity are shown as % IA/g tissue, representing the average ± SD (n = 3–4).

**Organs**	**[^{177}Lu]Lu-6*R*-RedFol-1**		**[^{177}Lu]Lu-6*S*-RedFol-1**		**[^{177}Lu]Lu-OxFol-1**	
	**1 h p.i.**	**1 h p.i. + FA**	**1 h p.i.**	**1 h p.i. + FA**	**1 h p.i.[1]**	**1 h p.i. + FA**
Blood	23 ± 3	25 ± 2	19 ± 2	23 ± 2	7.9 ± 1.4	20 ± 1
Kidneys	8.4 ± 0.8	4.9 ± 0.6	18 ± 2	7.0 ± 0.5	23 ± 4	9.2 ± 4.0
Liver	3.3 ± 0.3	4.1 ± 0.4	2.6 ± 0.1	3.7 ± 0.5	5.0 ± 1.3	3.3 ± 0.2
Salivary gl.	5.0 ± 0.3	5.3 ± 0.4	4.0 ± 0.2	4.6 ± 0.4	9.8 ± 2.4	3.9 ± 0.8
KB Tumor	11 ± 1	5.2 ± 0.5	14 ± 3	6.0 ± 0.9	11 ± 1	6.6 ± 0.7

([1] The data were reproduced from Siwowska et al., 2017 Mol Pharm 14:523 [1]. Copyright 2020 American Chemical Society).

9. SPECT/CT imaging studies

Purpose: SPECT/CT imaging studies were performed to visualize and compare the accumulation of the folate radioconjugates in KB tumors and normal tissues of mice.

Methods: Imaging studies were performed using a four-head, multiplexing, multipinhole small-animal SPECT camera (NanoSPECT/CT™, Mediso Medical Imaging Systems, Budapest, Hungary) as previously reported [1]. Each head was outfitted with a tungsten-based aperture of nine 1.4 mm-diameter pinholes and a thickness of 10 mm. CT scans of 7.5 min duration were followed by SPECT scans of ~40 min performed 4 h and 24 h after injection of the folate radioconjugates (25 MBq, 0.5 nmol, 100 µL, diluted in PBS containing 0.05% BSA). The images were acquired using Nucline Software (version 1.02, Mediso Ltd., Budapest, Hungary). The real-time CT reconstruction used a cone-beam filtered backprojection. The reconstruction of SPECT data was performed with HiSPECT software (version 1.4.3049, Scivis GmbH, Göttingen, Germany) using γ-energies of 56.1 keV (± 10%), 112.9 keV (± 10%) and 208.4 keV (± 10%) for lutetium-177. Images were prepared using VivoQuant post-processing software (version 3.5, inviCRO Imaging Services and Software, Boston, U.S.). A Gauss post-reconstruction filter (FWHM = 1 mm) was applied and the scale of activity was set as indicated on the images (minimum value = 2.5 Bq/voxel to maximum value = 50 Bq/voxel).

Results: The results of the SPECT/CT imaging study are reported in the main article.

10. Determination of areas under the curve (AUC)

Purpose: In order to compare the total uptake of the radioconjugates in the KB tumor, blood and kidneys, areas under the time-activity curves were integrated and compared.

Methods: The experimental setting and analysis of the non-decay corrected biodistribution data are described in the main article.

Results: The calculated $AUC_{0\text{-}120h}$ values and the tumor-to-background $AUC_{0\text{-}120h}$ ratios are summarized in Table S5. The data were analyzed for significance using a one-way ANOVA test with a Tukey's multiple comparisons post-test (GraphPad Prism software, version 7). A *p*-value of <0.05 was considered as statistically significant. The graphs and the table with $AUC_{0\text{-}120h}$ expressed as values relative to those previously obtained for [^{177}Lu]Lu-OxFol-1 [1] are reported in the main article.

Table S5 Area under the curve ($AUC_{0\text{-}120h}$) values (indicated as [(% IA/g)·h]) based on non-decay-corrected biodistribution data and tumor-to-background AUC ratios

	**$AUC_{0\to120h}$ Values**		
	**[^{177}Lu]Lu-6*R*-RedFol-1**	**[^{177}Lu]Lu-6*S*-RedFol-1**	**[^{177}Lu]Lu-OxFol-1[1]**
KB tumor	3332 ± 263*	3750 ± 232*	1041 ± 59
Blood	485 ± 20*	361 ± 21*	108 ± 6
Kidneys	1621 ± 118	5028 ± 230*	1799 ± 75
Liver	108 ± 5*	66 ± 5*	205 ± 24
	**$AUC_{0\to120h}$ Ratios**		
	**[^{177}Lu]Lu-6*R*-RedFol-1**	**[^{177}Lu]Lu-6*S*-RedFol-1**	**[^{177}Lu]Lu-OxFol-1[1]**
AUC_{Tu}-to-AUC_{Bl}	6.9 ± 0.8	10 ± 2	9.6 ± 1.1
AUC_{Tu}-to-AUC_{Ki}	2.1 ± 0.4*	0.7 ± 0.1	0.6 ± 0.1
AUC_{Tu}-to-AUC_{Li}	31 ± 4*	57 ± 8*	5.1 ± 0.9

* Significantly different (p<0.05) from the value of [^{177}Lu]Lu-OxFol-1

[1] Data for [^{177}Lu]Lu-OxFol-1 were previously published [1]

11. SPECT/CT imaging studies after blocking FRs with folic acid and cm13

Purpose: In order to determine unspecific (FR-unrelated) accumulation of the folate radioconjugates in tumors and kidneys, SPECT/CT imaging studies were performed with KB tumor-bearing mice after injection of excess folic acid or excess non-labeled cm13, an albumin-binding folic acid conjugate [1]. [^{177}Lu]Lu-DOTA-PPB-01, a radioligand consisting of the *p*-iodophenyl-based albumin binder and a DOTA-chelator [3], was also investigated in order to visualize potential unspecific accumulation of an albumin-binding radioligand without targeting agent.

Methods: SPECT/CT imaging studies to demonstrate the ability to block uptake of the folate radioconjugates in FR-positive tissues were performed with KB tumor-bearing mice (n = 2). These mice were pre-injected with excess folic acid (100 µg) or excess non-labeled albumin-binding folate, cm13

[1], and scanned at 4 h and 24 h after injection of the radioconjugate. An additional KB tumor-bearing mouse was injected with equal amounts of [^{177}Lu]Lu-DOTA-PPB-01 [3], in order to demonstrate the FR-unrelated accumulation of activity in tumors. The SPECT/CT scans were performed as described in the main article using the scanner settings and image preparation tools, as described above.

Results: The SPECT/CT images of KB tumor-bearing mice injected with folate radioconjugates only (Fig. S6a/e, S7a/e, S8a/e) or with excess folic acid (Fig. S6b/f, S7b/f, S8b/f) or excess cm13 (Fig. S6c/g, S7c/g, S8c/g), respectively, demonstrated that the uptake of the folate radioconjugates in tumors and kidneys was mostly FR-dependent. Due to the fast excretion of folic acid, the uptake of the radioconjugates in FR-positive tumors and kidneys became manifest again at later time points (24 h p.i.), in particular in the case of [^{177}Lu]Lu-6*S*-RedFol-1 (Fig. 7f) which accumulated to a high extent in tumors and kidneys (Fig. 7e). Pre-injection of cm13, an albumin-binding folate conjugate with similar kinetics as the herein described folate radioconjugates, resulted in almost entire blockade of [^{177}Lu]Lu-6*S*-RedFol-1 accumulation in tumors and kidneys, respectively (Fig. 7g). The images acquired of a mouse that was injected with [^{177}Lu]Lu-DOTA-PPB-01 (Fig. S6d/h, S7d/h, S8d/h) demonstrated only low accumulation of activity in the tumors, which can be ascribed to an unspecific uptake mechanism. The extent of uptake in the tumors corresponded relatively well with residual activity in tumors of mice pre-injected with folic acid or cm13, indicating that it was caused by a FR-unrelated mechanism.

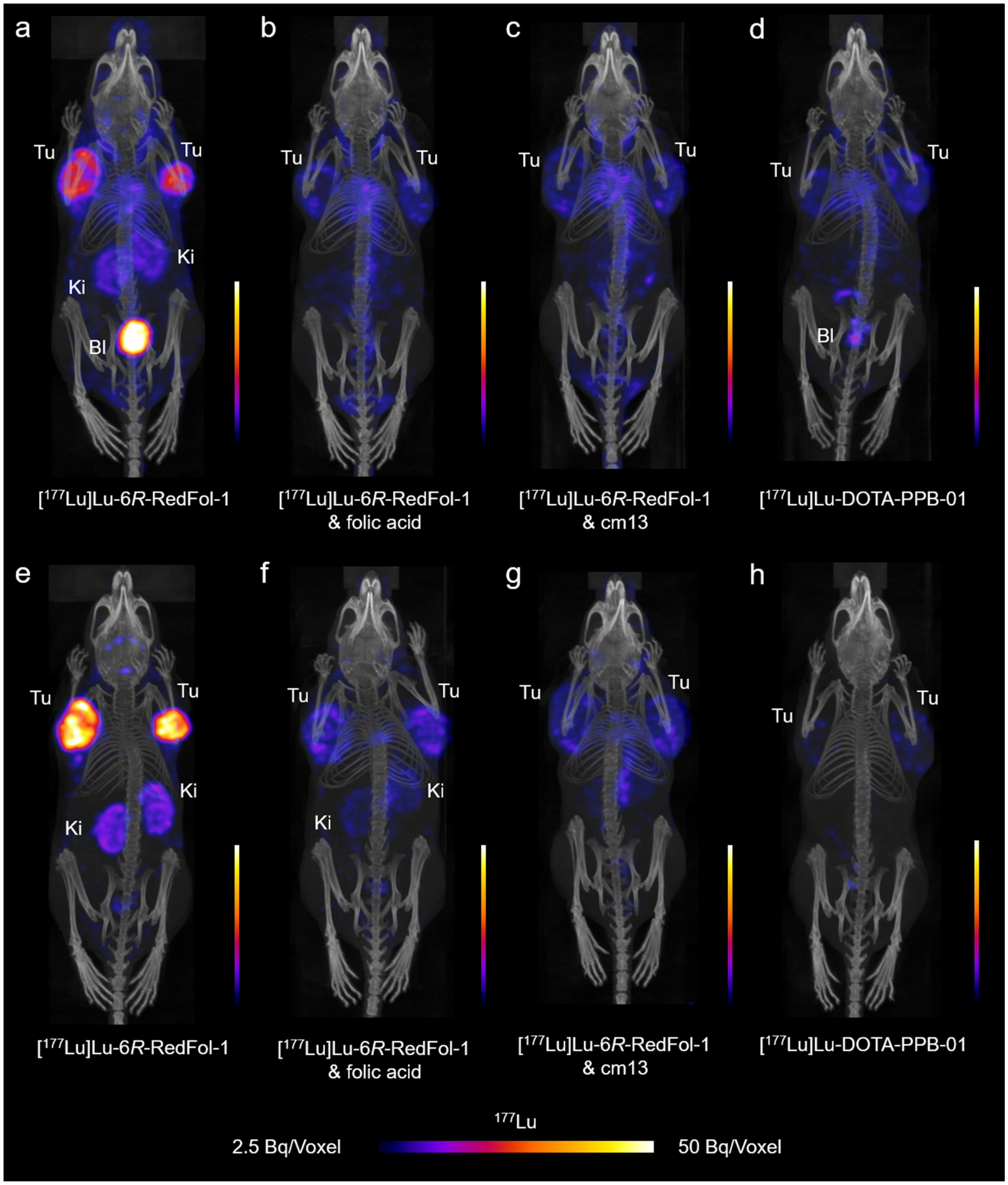

Fig. S6 SPECT/CT images shown as maximum intensity projections (MIPs) of KB tumor-bearing mice at 4 h p.i. (**a–d**) and 24 h p.i. (**e–h**). (**a**/**e**) Mouse injected with [^{177}Lu]Lu-6*R*-RedFol-1 (25 MBq; 0.5 nmol per mouse) only; (**b**/**f**) Mouse pre-injected with folic acid (100 µg); (**c**/**g**) Mouse pre-injected with cm13 (100 µg). (**d**/**h**) Mouse injected with [^{177}Lu]Lu-DOTA-PPB-01. Tu = KB tumor; Ki = kidney; Bl = urinary bladder.

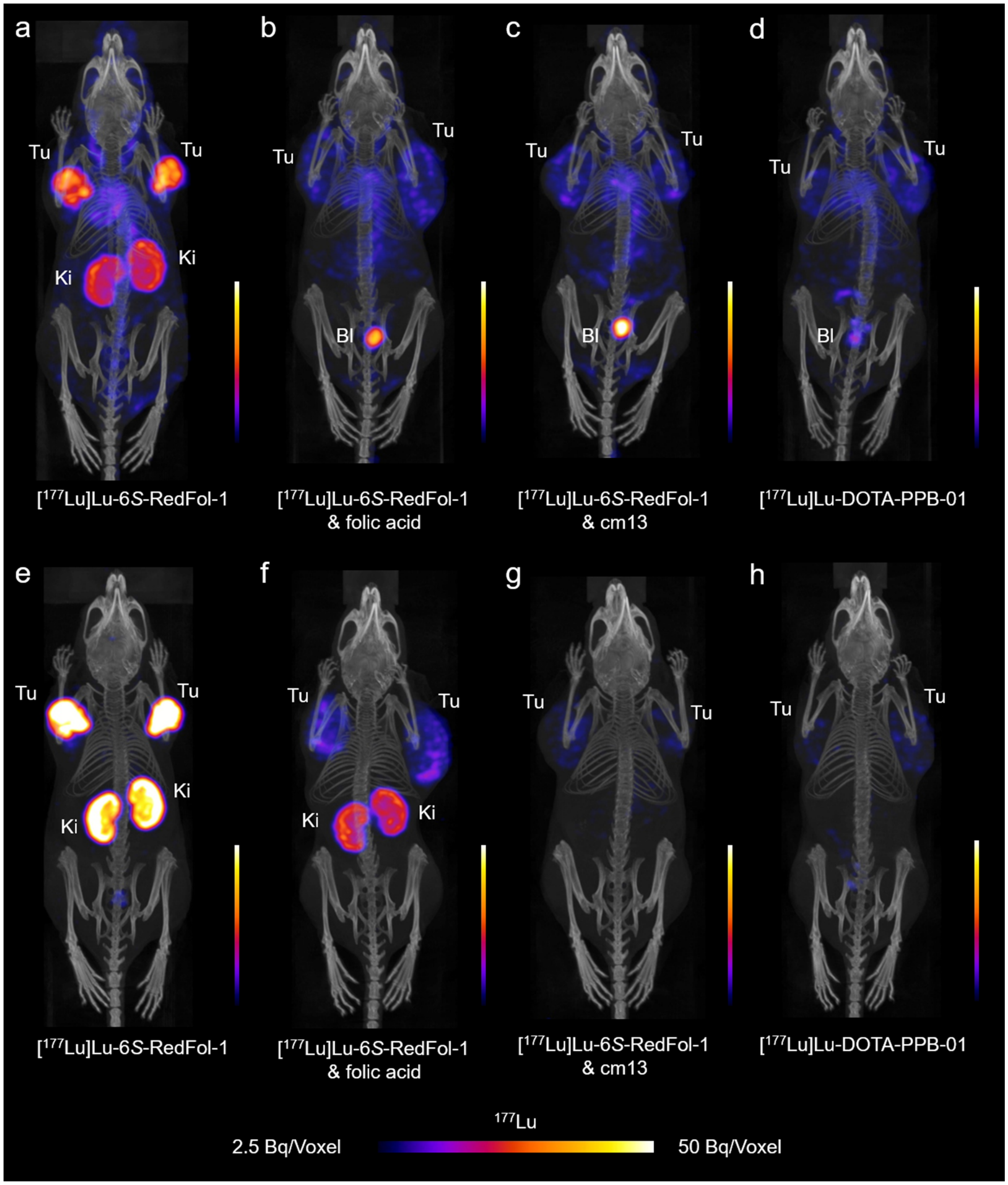

Fig. S7 SPECT/CT images shown as maximum intensity projections (MIPs) of KB tumor-bearing mice at 4 h p.i. (**a–d**) and 24 h p.i. (**e–h**). (**a**/**e**) Mouse injected with [^{177}Lu]Lu-6*S*-RedFol-1 (25 MBq; 0.5 nmol per mouse) only; (**b**/**f**) Mouse pre-injected with folic acid (100 µg); (**c**/**g**) Mouse pre-injected with cm13 (100 µg). (**d**/**h**) Mouse injected with [^{177}Lu]Lu-DOTA-PPB-01. Tu = KB tumor; Ki = kidney; Bl = urinary bladder.

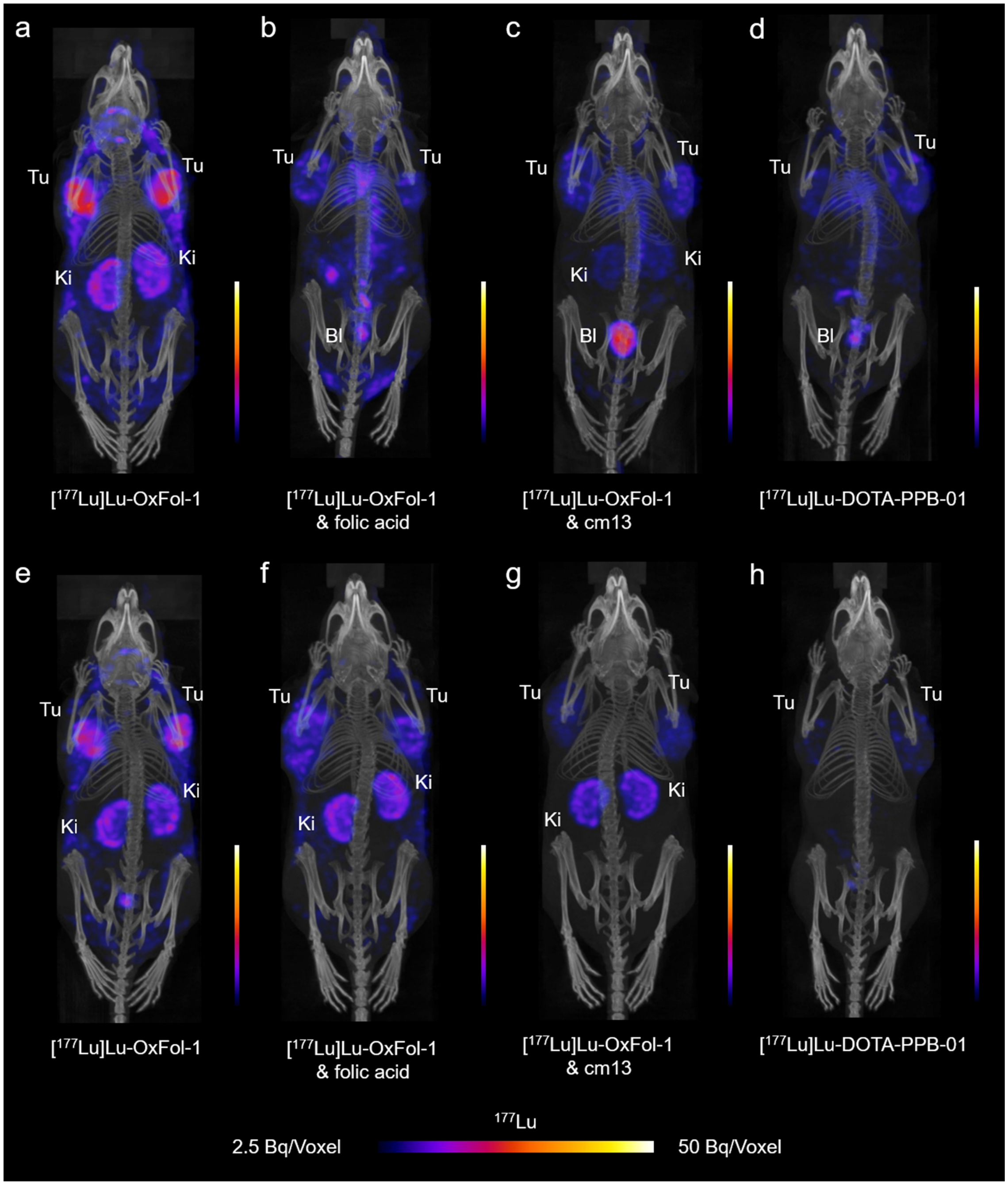

Fig. S8 SPECT/CT images shown as maximum intensity projections (MIPs) of KB tumor-bearing mice at 4 h p.i. (**a–d**) and 24 h p.i. (**e–h**). (**a/e**) Mouse injected with [^{177}Lu]Lu-OxFol-1 (25 MBq; 0.5 nmol per mouse) only; (**b/f**) Mouse pre-injected with folic acid (100 µg); (**c/g**) Mouse pre-injected with cm13 (100 µg). (**d/h**) Mouse injected with [^{177}Lu]Lu-DOTA-PPB-01. Tu = KB tumor; Ki = kidney; Bl = urinary bladder.

11. Assessment of the therapy study: Determination of tumor growth indices ($TGDI_x$)

Purpose: The therapy study was performed to demonstrate the effect of [^{177}Lu]Lu-6*R*-RedFol-1 and to compare the results with those obtained with [^{177}Lu]Lu-OxFol-1 applied at equal activities.

Methods: The $TGDI_x$ values were calculated and analyzed for significance using a one-way ANOVA test with a Tukey's multiple comparisons post-test (GraphPad Prism software, version 7). A *p*-value of <0.05 was considered as statistically significant.

Results: The $TGDI_x$ values and significance analysis are listed in Tables S6/S7.

Table S6 Tumor growth delay index with x-fold increase of tumor size ($TGDI_x$) of [^{177}Lu]Lu-6*R*-RedFol-1 and [^{177}Lu]Lu-OxFol-1

Group (n=6–9)	Treatment	$TGDI_2$	$TGDI_5$	$TGDI_8$
A	0.05% BSA/PBS	1.0 ± 0.4	1.0 ± 0.3	1.0 ± 0.2
B	[^{177}Lu]Lu-6*R*-RedFol-1 (10 MBq)	4.2 ± 0.6	2.5 ± 0.3	2.2 ± 0.2
C	[^{177}Lu]Lu-OxFol-1 (10 MBq)	2.4 ± 0.3	1.5 ± 0.2	1.5 ± 0.3
D	[^{177}Lu]Lu-6*R*-RedFol-1 (15 MBq)[1]	n.d.	n.d.	n.d.
E	[^{177}Lu]Lu-OxFol-1 (15 MBq)	4.5 ± 0.3	2.6 ± 0.3	2.4 ± 0.3

[1] $TGDI_x$ of Group D was not determined (n.d.) due to RTV < 2

Table S7 Statistical significance of difference between Groups A–E in $TGDI_x$ values tested using one-way ANOVA with a Tukey's multiple comparisons post-test

Compared groups	$TGDI_2$	$TGDI_5$	$TGDI_8$
	p-values		
A vs. B	<0.05	<0.05	<0.05
A vs. C	<0.05	<0.05	<0.05
A vs. D	n.d.	n.d.	n.d.
A vs. E	<0.05	<0.05	<0.05
B vs. C	<0.05	<0.05	<0.05
B vs. D	n.d.	n.d.	n.d.
B vs. E	>0.05	>0.05	>0.05
C vs. D	n.d.	n.d.	n.d.
C vs. E	<0.05	<0.05	<0.05

n.d. = not determined

12. Assessment of the therapy study: Potential side effects

Purpose: In order to assess potentially undesired side effects of [^{177}Lu]Lu-6*R*-RedFol-1 and [^{177}Lu]Lu-OxFol-1 therapy, the relative body weights, organ mass-to-brain ratios and organ mass-to-body weight ratios as well as blood plasma parameters were determined and compared.

Methods:

Relative body weight, organ mass and mass ratios: Mice were euthanized when a predefined endpoint criterion was reached (described in the main article) or when the study was terminated at Day 56. Body weight and organ mass ratios (kidney-to-brain, liver-to-brain and spleen-to-brain) were analyzed for significance using a one-way ANOVA test with a Dunnett's multiple comparisons post-test (GraphPad Prism software, version 7). A p-value of <0.05 was considered as statistically significant.

Blood plasma chemistry: Immediately before euthanasia of the mice that reached the endpoint, blood was sampled from the retrobulbar vein. The values of creatinine (CRE), blood urea nitrogen (BUN), alkaline phosphatase (ALP), total bilirubin (TBIL) and albumin (ALB) were determined in the plasma after centrifugation of the blood using a dry chemistry analyzer (DRI-CHEM 4000i, FUJIFILM, Japan). The average blood plasma parameters of each group were analyzed for significance using a one-way ANOVA test with a Dunnett's multiple comparisons post-test (GraphPad Prism software, version 7). A p-value of <0.05 was considered as statistically significant.

Necropsy, tissue processing and histological examination: After euthanasia kidneys and spleen were weighted, fixed in 4% neutral-buffered formalin and routinely embedded in paraffin wax. Formalin fixed sternum and femur were decalcified at room temperature in an ethylenediaminetetraacetic acid (EDTA)-citrate solution for 48 hours before embedding in paraffin wax. Sections of 3–5 μm thickness were prepared on glass slides and routinely stained with hematoxylin eosin (HE). Histological lesions in the kidneys, spleen and bone marrow were semi-quantitatively scored by a veterinary pathologist in a blind manner as previously described in Haller et al. 2016 and Siwowska et al. 2019 [4, 5].

Results:

Relative body weight, organ mass and mass ratios: The relative body weight of treated mice measured at Day 14 and at the endpoint was comparable to the control group in most of the cases (p>0.05). Exceptions were the values of Group C at Day 14 and Group D at the endpoint which were lower and higher, respectively, compared to the control group (p<0.05) (Table S8).

Table S8 Relative body weight of mice at Day 14 and at the endpoint of the therapy study

**Group** (n=6–9)	**Relative body weight at Day 14**[1] (average ± SD)	**Relative body weight**[2] (average ± SD)
A	1.08 ± 0.05	1.10 ± 0.06
B	1.05 ± 0.04	1.08 ± 0.03
C	1.00 ± 0.04*	1.05 ± 0.07
D	1.08 ± 0.03	1.21 ± 0.06*
E	1.06 ± 0.04	1.16 ± 0.06

* Significantly different ($p<0.05$) from the value of Group A

[1] Data obtained at Day 14 when the first control mouse reached an endpoint

[2] Data obtained at the day of euthanasia when an endpoint criterion was reached or at the end of the study

The mass of kidneys, liver, spleen and brain in Group D and E, as well as the respective organ-to-brain and organ-to-body weight ratios, were lower than the organ masses of the mice of Group A ($p<0.05$) (Tables S9/S10). It is worth mentioning, however, that not all mice were euthanized at the same time, which explain the discrepancy in the measured kidney and spleen masses.

Table S9 Organ mass of mice of the therapy study collected after euthanasia

**Group** (n=6–9)	**Organ mass**[1] (mg) (average ± SD)			
	Kidneys	Liver	Spleen	Brain
A	379 ± 41	1277 ± 139	137 ± 20	457 ± 23
B	370 ± 30	1269 ± 76	119 ± 31	469 ± 21
C	375 ± 48	1367 ± 171	140 ± 16	466 ± 22
D	287 ± 18*	1237 ± 101	58 ± 5*	452 ± 17
E	297 ± 27*	1096 ± 112	85 ± 8*	432 ± 35

* Significantly different ($p<0.05$) from the value of Group A

[1] Data obtained at the day of euthanasia when an endpoint criterion was reached or at the end of the study

Table S10 Organ mass-to-brain mass and organ mass-to-body weight ratios

**Group** (n=6–9)	**Organ mass-to-brain mass ratios** (average ± SD)		
	Kidney-to-brain	Liver-to-brain	Spleen-to-brain
A	0.83 ± 0.07	2.8 ± 0.2	0.31 ± 0.05
B	0.79 ± 0.05	2.7 ± 0.1	0.25 ± 0.06*
C	0.81 ± 0.10	2.9 ± 0.4	0.30 ± 0.04
D	0.64 ± 0.05*	2.7 ± 0.2	0.13 ± 0.01*
E	0.69 ± 0.05*	2.5 ± 0.2	0.20 ± 0.02*
**Group** (n=6–9)	**Organ mass-to-body weight ratios** (average ± SD)		
	Kidney-to-body	Liver-to-body	Spleen-to-body
A	0.014 ± 0.001	0.047 ± 0.003	0.005 ± 0.001
B	0.014 ± 0.001	0.048 ± 0.003	0.004 ± 0.001*
C	0.014 ± 0.001	0.052 ± 0.004*	0.005 ± 0.001
D	0.012 ± 0.001*	0.051 ± 0.002	0.002 ± 0.000 *
E	0.012 ± 0.001*	0.045 ± 0.003	0.003 ± 0.000*

* Significantly different ($p<0.05$) from the value of Group A

Blood plasma chemistry: Apart from the albumin plasma concentrations in Group D ($p<0.05$), no significant differences were observed between the untreated mice and mice treated with [^{177}Lu]Lu-6*R*-RedFol-1 and [^{177}Lu]Lu-OxFol-1 at the endpoint of the therapy (Table S11).

Table S11 Plasma chemistry determined at the endpoint of the therapy (n=6, if not differently indicated)

**Group**	**CRE** (µmol/L)	**BUN** (mmol/L)	**ALP** (U/L)	**TBIL** (µmol/L)	**ALB** (g/L)
A	<18 (n=9)	5.81 ± 0.78 (n=9)	82 ± 16 (n=9)	<3 (n=9)	21 ± 1 (n=9)
B	<18 (n=5)<br>18 (n=1)	7.09 ± 0.90	77 ± 14	<3 (n=4)<br>3 ± 0 (n=2)	21 ± 1
C	<18	7.43 ± 1.30	90 ± 10	<3 (n=5)<br>4 (n=1)	22 ± 2
D	<18	6.81 ± 0.27	83 ± 15	<3 (n=2)<br>4 ± 1 (n=4)	23 ± 1*
E	<18	6.96 ± 1.84	95 ± 12	<3 (n=4)<br>3 ± 0 (n=2)	22 ± 1

* Significantly different ($p<0.05$) from the value of Group A

Necropsy, tissue processing and histological examination: Histological investigations did not reveal any pathological changes in kidneys, spleen and bone marrow that could be attributed to the treatment with [^{177}Lu]Lu-6*R*-RedFol-1. No remarkable histological differences were observed between [^{177}Lu]Lu-6*R*-RedFol-1 mice and animals treated with [^{177}Lu]Lu-OxFol-1 (Table S12).

Table S12 Histopathological scoring of kidneys, bone marrow and spleen collected from KB tumor-bearing mice which received [^{177}Lu]Lu-6*R*-RedFol-1 or [^{177}Lu]Lu-OxFol-1 at 10 MBq or 15 MBq activity level.

**Folate Radioconjugate**	**Activity**	**Kidneys**	**Spleen**		**Bone Marrow**
		Radiation nephropathy	White pulp depletion	Extramedullary haematopoiesis	Hypocellularity
- (0.05% BSA/PBS)	0	0 (9/9)	0 (9/9)	1 (1/9)<br>2 (1/9)<br>3 (7/9)	0 (9/9)
Average		0.0	0.0	2.7	0.0
[^{177}Lu]Lu-6*R*-RedFol-1	10 MBq	0 (6/6)	0 (5/6)<br>1 (1/6)	1 (1/6)<br>2 (4/6)<br>3 (1/6)	0 (4/6)<br>1 (2/6)
Average		0.0	0.2	2.0	0.3
[^{177}Lu]Lu-OxFol-1	10 MBq	0 (6/6)	0 (4/6)<br>1 (2/6)	3 (6/6)	0 (6/6)
Average		0.0	0.3	3.0	0.0
[^{177}Lu]Lu-6*R*-RedFol-1	15 MBq	0 (5/6)<br>1 (1/6)	0 (6/6)	1 (3/6)<br>2 (3/6)	0 (3/6)<br>1 (3/6)
Average		0.2	0.0	1.5	0.5
[^{177}Lu]Lu-OxFol-1	15 MBq	0 (6/6)	0 (3/6)<br>1 (3/6)	2 (2/6)<br>3 (4/6)	0 (6/6)
Average		0.0	0.5	2.7	0.0

References

1. Siwowska K, Haller S, Bortoli F, Benesova M, Groehn V, Bernhardt P, et al. Preclinical comparison of albumin-binding radiofolates: impact of linker entities on the in vitro and in vivo properties. Molecular pharmaceutics. 2017;14:523-32. doi:10.1021/acs.molpharmaceut.6b01010.
2. Umbricht CA, Benesova M, Schibli R, Müller C. Preclinical development of novel PSMA-targeting radioligands: modulation of albumin-binding properties to improve prostate cancer therapy. Molecular pharmaceutics. 2018;15:2297-306. doi:10.1021/acs.molpharmaceut.8b00152.
3. Müller C, Farkas R, Borgna F, Schmid RM, Benesova M, Schibli R. Synthesis, radiolabeling, and characterization of plasma protein-binding ligands: potential tools for modulation of the pharmacokinetic properties of (radio)pharmaceuticals. Bioconjugate chemistry. 2017;28:2372-83. doi:10.1021/acs.bioconjchem.7b00378.
4. Haller S, Pellegrini G, Vermeulen C, van der Meulen NP, Köster U, Bernhardt P, et al. Contribution of Auger/conversion electrons to renal side effects after radionuclide therapy: preclinical comparison of ^{161}Tb-folate and ^{177}Lu-folate. EJNMMI research. 2016;6:13. doi:10.1186/s13550-016-0171-1.
5. Siwowska K, Guzik P, Domnanich KA, Monne Rodriguez JM, Bernhardt P, Ponsard B, et al. Therapeutic potential of ^{47}Sc in comparison to ^{177}Lu and ^{90}Y: preclinical investigations. Pharmaceutics. 2019;11. doi:10.3390/pharmaceutics11080424.
